# Lack of inflammatory gene expression in bats: a unique role for a transcription repressor

**DOI:** 10.1038/s41598-017-01513-w

**Published:** 2017-05-22

**Authors:** Arinjay Banerjee, Noreen Rapin, Trent Bollinger, Vikram Misra

**Affiliations:** 1 Department of Veterinary Microbiology, Western College of Veterinary Medicine, University of Saskatchewan, Saskatoon, Saskatchewan S7N 5B4 Canada; 2 Department of Veterinary Pathology, Western College of Veterinary Medicine, University of Saskatchewan, Saskatoon, Saskatchewan S7N 5B4 Canada

## Abstract

In recent years viruses similar to those that appear to cause no overt disease in bats have spilled-over to humans and other species causing serious disease. Since pathology in such diseases is often attributed to an over-active inflammatory response, we tested the hypothesis that bat cells respond to stimulation of their receptors for viral ligands with a strong antiviral response, but unlike in human cells, the inflammatory response is not overtly activated. We compared the response of human and bat cells to poly(I:C), a viral double-stranded RNA surrogate. We measured transcripts for several inflammatory, interferon and interferon stimulated genes using quantitative real-time PCR and observed that human and bat cells both, when stimulated with poly(I:C), contained higher levels of transcripts for interferon beta than unstimulated cells. In contrast, only human cells expressed robust amount of RNA for TNFα, a cell signaling protein involved in systemic inflammation. We examined the bat TNFα promoter and found a potential repressor (c-Rel) binding motif. We demonstrated that c-Rel binds to the putative c-Rel motif in the promoter and knocking down c-Rel transcripts significantly increased basal levels of TNFα transcripts. Our results suggest bats may have a unique mechanism to suppress inflammatory pathology.

## Introduction

Bats are thought to be natural reservoirs for several emerging and re-emerging viruses such as those that closely resemble severe acute respiratory syndrome (SARS), Middle East respiratory syndrome (MERS) and porcine epidemic diarrhoea (PED) – causing coronaviruses (CoV), Marburg and, possibly, Ebola filoviruses, and Hendra and Nipah paramyxoviruses, amongst others^1–5^. These viruses are speculated to have spilled over from bats to humans and other animals, directly or through intermediate hosts, causing severe and often fatal disease. Despite evidence of bats harbouring these viruses, or viruses closely related to them, bats do not appear to show overt symptoms or clinical signs of infection^6^. Infecting Pteropid, Jamaican and Egyptian fruit bats with Nipah and Hendra viruses, MERS-CoV and Ebolavirus yielded no evidence of disease. The bats sero-converted and in some cases virus could be detected post infection^7–10^, but these bats did not demonstrate signs of illness. We do not completely understand why bats are less susceptible to these viral infections than other mammals that often succumb.

The immune system, based on our knowledge from humans and other mammals, can be broadly categorised into two branches – the innate immune system and the adaptive immune system^11^. Both branches are distinct, although there is interaction between them. During viral infection, the innate response is the first line of defence and primes the adaptive immune response against the virus^12, 13^. A virus infected cell detects several pathogen associated molecular patterns (PAMPs) associated with the virus through pattern recognition receptors (PRRs) present in endosomal compartments, cytoplasm and cell membrane [reviewed by Mogensen^14^]. Some of these PRRs, such as toll-like receptors (TLRs) 3, 7, 8, 9, Retinoic acid-inducible gene I (RIG-I) and Melanoma Differentiation-Associated protein 5 (MDA5), have specifically evolved to recognise microbial nucleic acids [reviewed by Lee and Kim^15^]. Polyinosinic:polycytidylic acid [poly(I:C)] is a known double-stranded RNA analogue which is detected by TLR3, RIG-I and MDA5. After detection, PRRs signal through mediators to activate two pathways - the antiviral cytokine (interferons) and inflammatory pathways^16^.

**Figure 1:**
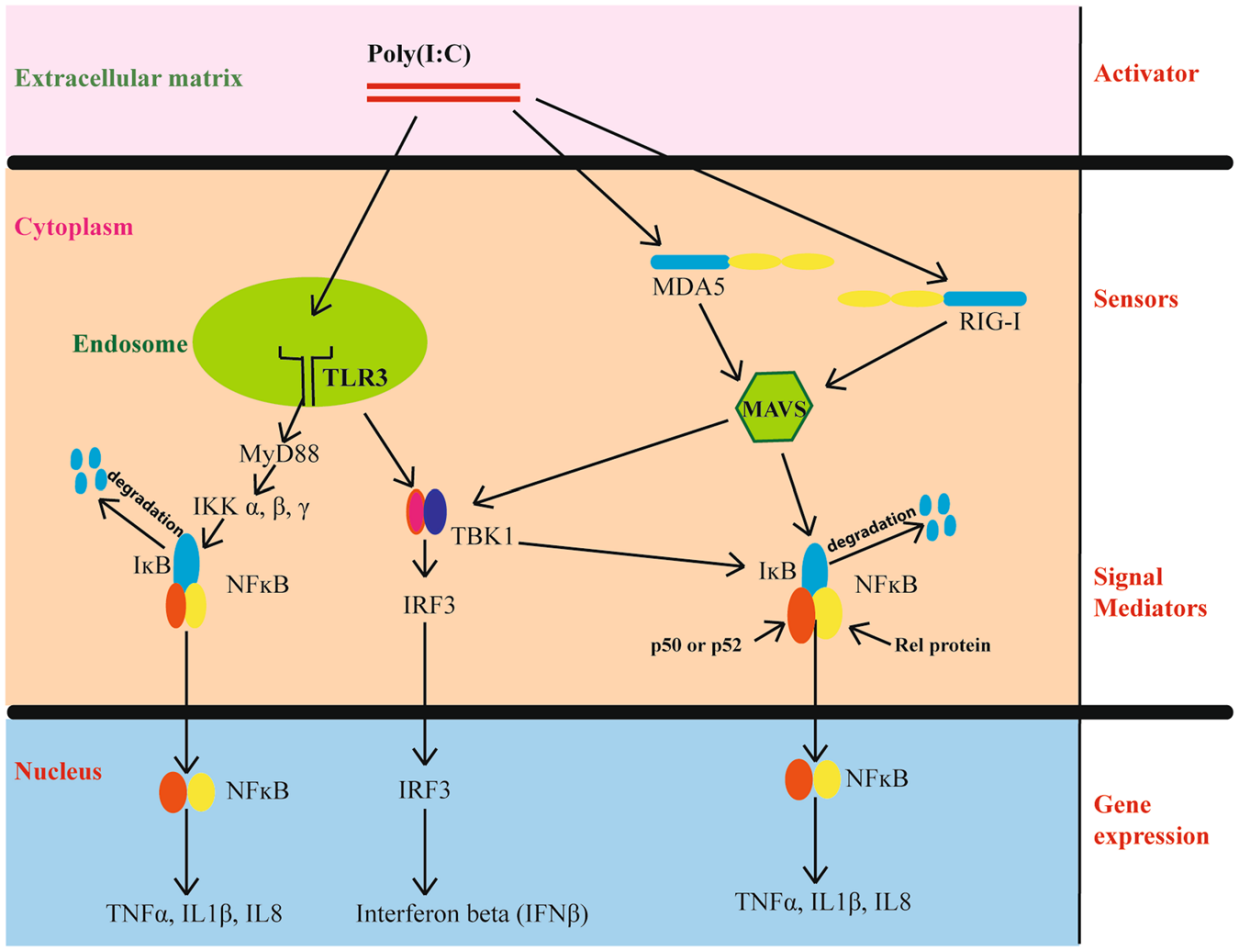
Schematic representation of detection of double-stranded RNA in a human cell and activation of the innate immune response. RNA viruses during replication produce double-stranded RNA intermediates (PAMPs), which are detected by cellular receptors (PRRs). Poly(I:C) is a known double-stranded RNA analogue (activator) which is detected by sensors such as TLR3 (black), RIGI and MDA5 (blue, CARD domains in yellow) in a cell. These sensors, when stimulated by the activator, lead to the expression of interferons (IFNβ) and inflammatory genes (TNFα, IL1β, IL8) through adaptor proteins (MAVS and MyD88) and signal mediators such as NFκB (orange and yellow subunits) and IRF3. NFκB is retained in an inactive state in the cytoplasm by inhibitory molecules such as IκB (blue). Upon receiving an activation signal via a sensor, kinases (TBK1) phosphorylate IRF3, which then translocates to the nucleus to activate transcription. Kinases, such as IKK α, β or γ phosphorylate IκB inhibitors and mark them for degradation, thereby activating NFκB. Active NFκB then causes expression of downstream genes by translocating to the nucleus.

Nuclear factor kappa-light-chain-enhancer of activated B cells (NFκB) and interferon regulatory factor 3 (IRF3) are two signal mediators that activate antiviral and inflammatory pathways in response to double-stranded RNA sensed by TLR3, RIG-I and MDA5 [reviewed by Mogensen^14^]. Five members of the NFκB family of proteins have been identified in humans, namely, RelA (p65), RelB, c-Rel, NFκB-1 (p50) and NFκB-2 (p52). All five members form homo- or hetero-dimers and share some structural features. These dimers are bound by molecules of the inhibitor of NFκB (IκB) family and retained in the cytoplasm of the cell in an inactivated state. After PAMP recognition, downstream signals mark the inhibitors for degradation and the dimers translocate to the nucleus of the cell to cause expression of antiviral and inflammatory genes^17^ (Fig. 1). Different combinations of the proteins have vastly different effects on gene expression^18^. For instance, hetero-dimers of p50 or p52 and p65 or RelB activate transcription. In contrast, c-Rel as a homo-dimer or in association with p50 or p65, represses transcriptional activation by NFκB^19^.

*Chiroptera* is a very diverse order and information about one genus or species may not apply to all bats. However, *Pteropus alecto* (black flying fox) is being extensively studied to better understand the bat immune system. Three and a half percent of *P*. *alecto* transcribed genes, amounting to about 500 genes, correspond to immune genes^20^. *P*. *alecto* homologs to human TLR 1–10 have been sequenced and TLR 13 has been described. RIG-I, major histocompatibility complex I (MHC-I) and interferon regulatory factor 7 (IRF7) have been detected and characterized^21–23^. The interferon pathway, immunoglobulins and the presence of microRNAs have been substantiated in this bat. Constitutive expression of interferon alpha and the ability of cells derived from *P*. *alecto* to mount an interferon beta (IFNβ) response to viral challenges has been demonstrated^24–30^.

A robust antiviral and a controlled inflammatory response is desirable to control a viral infection. During SARS-CoV and MERS-CoV infection in humans and PED-CoV infection in pigs, the viruses inhibit an early interferon response and cause massive secretion of pro-inflammatory chemokines and cytokines, leading to excessive recruitment of immune cells^31–33^. This is detrimental as an excessive inflammatory response causes tissue damage and organ dysfunction in the host^34^.

In this study, we hypothesized that *Eptesicus fuscus* (big brown bat) cells would mount a strong antiviral cytokine response but a low inflammatory response to poly(I:C), synthetic single-stranded RNA (ssRNA, a viral single-stranded RNA surrogate), and CpG oligo deoxynucleotides (CpG ODN, a viral and bacterial DNA surrogate). These are known stimulants for human TLRs 3, 7/8 and 9 respectively. We compared the response of immortalized *E*. *fuscus* kidney cells (Efk3)^35^ as well as *E*. *fuscus* bone marrow derived myeloid cells stimulated with poly(I:C) with that of human fibroblast cells (MRC5). We quantified the expression of innate response genes including IFNβ, tumor necrosis factor alpha (TNFα), interleukin 8 (IL8) and others using quantitative real-time polymerase chain reaction (qRT-PCR). We observed that both bat and human cells mounted a strong IFNβ response but only human cells expressed high levels of transcripts for proinflammatory cytokines such as TNFα and IL8 after TLR ligand treatments. To further explore the low TNFα response in bat cells, we analyzed the *E*. *fuscus* TNFα promoter for transcription factor binding motifs and identified a potential binding site for c-Rel proto-oncoprotein, a known suppressor of gene expression^36^. Ectopically expressed c-Rel bound to DNA containing this motif and the protein localized to the nucleus of bat cells in response to poly(I:C). Deletion of this motif in the promoter enhanced activation by poly(I:C) and partial knockdown of bat c-Rel RNA by specific small interfering RNA (siRNA) increased basal levels of TNFα transcripts in bat cells. We could detect c-Rel transcripts in every major big brown bat tissue, such as spleen, gut, ileum, kidney, lung, liver and the bat kidney cell line, unlike in humans, where it is found predominantly in hematopoetic cells^37^. Finally, we could also demonstrate that bat c-Rel bound to the potential motif as promoters containing the motif were co-immunoprecipitated to higher levels than promoters that lacked this motif. Our results suggest that bats might have evolved a unique mechanism to suppress an exaggerated inflammatory response to viruses.

## Results

### TLR expression in MRC5 and Efk3 cells

To determine if the human and bat cell lines we studied expressed receptors for viral ligands, we examined these cells for TLR 2, 3, 7, 8, 9, RIG-I and MDA5 using PCR (see Supplementary Table S1). Both cell lines contained transcripts for these receptors.

### Big brown bat cells express high levels of IFNβ but low TNFα transcripts in response to poly(I:C)

To determine if the cells were capable of innate responses to viral ligands we treated the cells with poly(I:C), single-stranded RNA (ssRNA) and CpG ODNs. We used these surrogates instead of viruses capable of infecting both cells to prevent modulation of these pathways by viral proteins.

**Figure 2:**
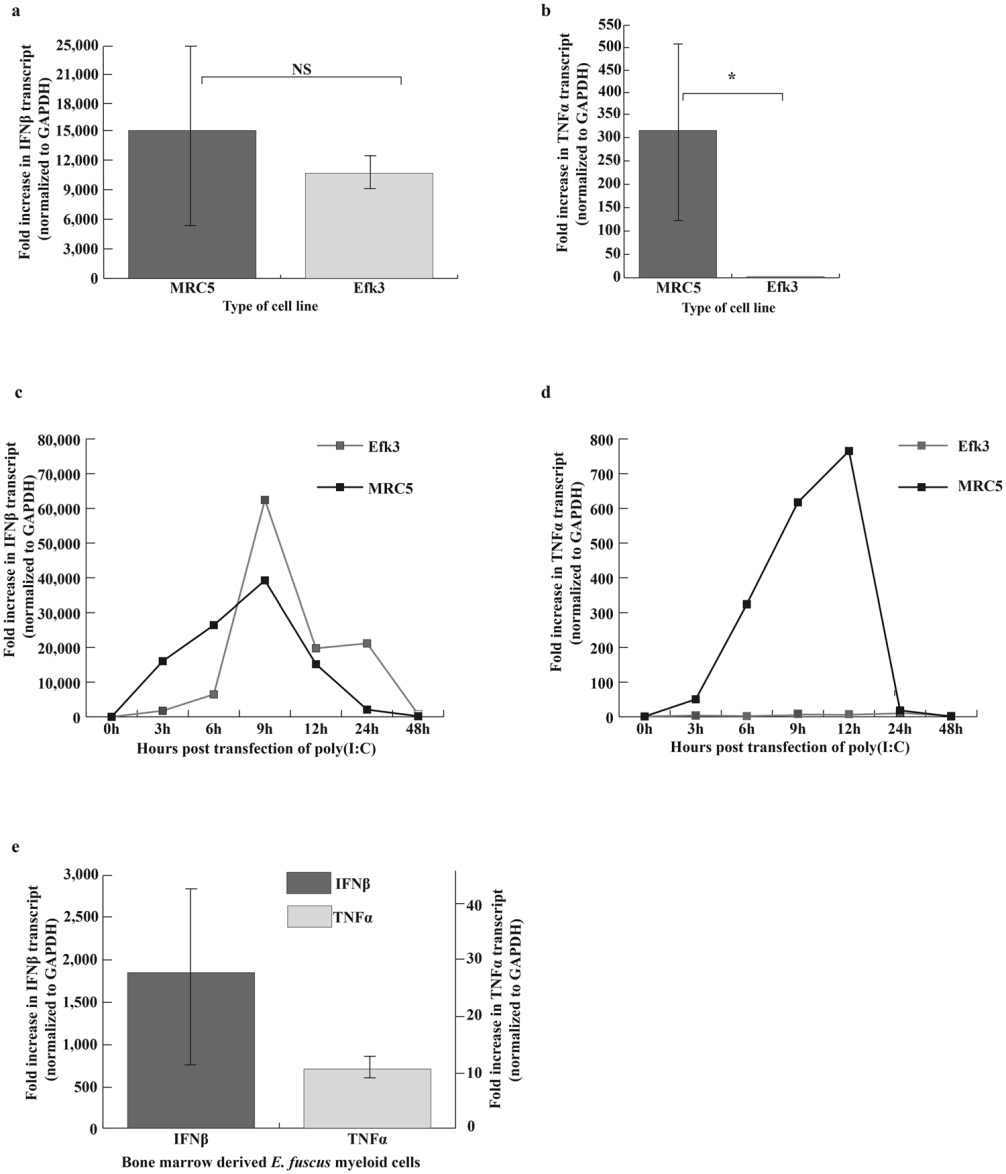
Efk3 cells do not express high levels of TNFα transcripts in response to poly(I:C). We transfected human fibroblasts, bat kidney (Efk3) and bat myeloid cells with poly(I:C), a known TLR3 stimulant, and studied the expression of IFNβ and TNFα relative to mock transfected cells. (**a**) Both MRC5 and Efk3 cells responded to poly(I:C) by expressing IFNβ transcripts (mean ± SD, n = 3, P = 0.05). (**b**) MRC5 cells responded to poly(I:C) by several hundred fold expression of TNFα transcripts but Efk3 cells expressed significantly lower levels of TNFα transcripts (mean ± SD, n = 3, P = 0.021). (**c**) Transcripts for IFNβ in MRC5 and Efk3 cells were quantified at several time points after poly(I:C) treatment. Both MRC5 and Efk3 cells showed highest IFNβ transcript levels 9 h post poly(I:C) treatment. (**d**) Transcripts for TNFα in MRC5 and Efk3 cells were quantified at several time points after poly(I:C) treatment. MRC5 cells showed highest TNFα transcripts 12 h post poly(I:C) treatment but Efk3 cells did not express TNFα transcripts to relatively comparable levels. (**e**) Big brown bat bone marrow derived myeloid cells expressed IFNβ transcripts to 1700 fold higher post poly(I:C) treatment but TNFα transcripts were expressed to an average of only 11 fold higher over mock treated cells (mean ± SD, n = 3). Results are expressed as fold increases over mock-treated cells normalized to GAPDH values (Materials and Methods). Statistical significance was calculated using two-tailed Mann-Whitney *U* test for two independent samples. *P < 0.05, NS = not significant. n is the number of independent experiments. SD = standard deviation.

We quantified the expression of several transcription factors and downstream genes by qRT-PCR. We observed a heightened innate response with poly(I:C) in bat cells but not as much with ssRNA and CpG ODN (see Supplementary Table S2). We therefore decided to further analyse the cytokine response in bat and human cells to poly(I:C). While both MRC5 and Efk3 cells responded to poly(I:C) with a robust increase in IFNβ transcripts (Fig. 2a), only MRC5 cells responded with increased levels of TNFα RNA (Fig. 2b). Poly(I:C)-treated Efk3 cells contained, on an average, 2.4 fold more TNFα transcripts than mock-treated cells, as compared to a 315-fold increase in human cells (Fig. 2b). We therefore examined further the response of these two genes. To determine when IFNβ and TNFα transcripts are expressed following poly(I:C) treatment, we quantified IFNβ and TNFα transcripts in MRC5 and Efk3 cells at different times after poly(I:C) treatment. Both MRC5 and Efk3 cells showed highest IFNβ transcript levels at 9 h post-transfection (Fig. 2c). TNFα transcript levels were highest at 12 h post-transfection in MRC5 cells and there was relatively little expression in Efk3 cells (Fig. 2d). To rule out the possibility of Efk3 cells not being able to mount a TNFα response, we transfected bone marrow derived myeloid cells from big brown bat long bones with poly(I:C). The mixed population of cells (see Supplemental Fig. S1) demonstrated an average of 1700-fold increase in IFNβ transcripts but only 11-fold increase in TNFα transcripts (Fig. 2e) post stimulation.

### Poly(I:C) signals through TLR3 to activate IFNβ in Efk3 cells

**Figure 3:**
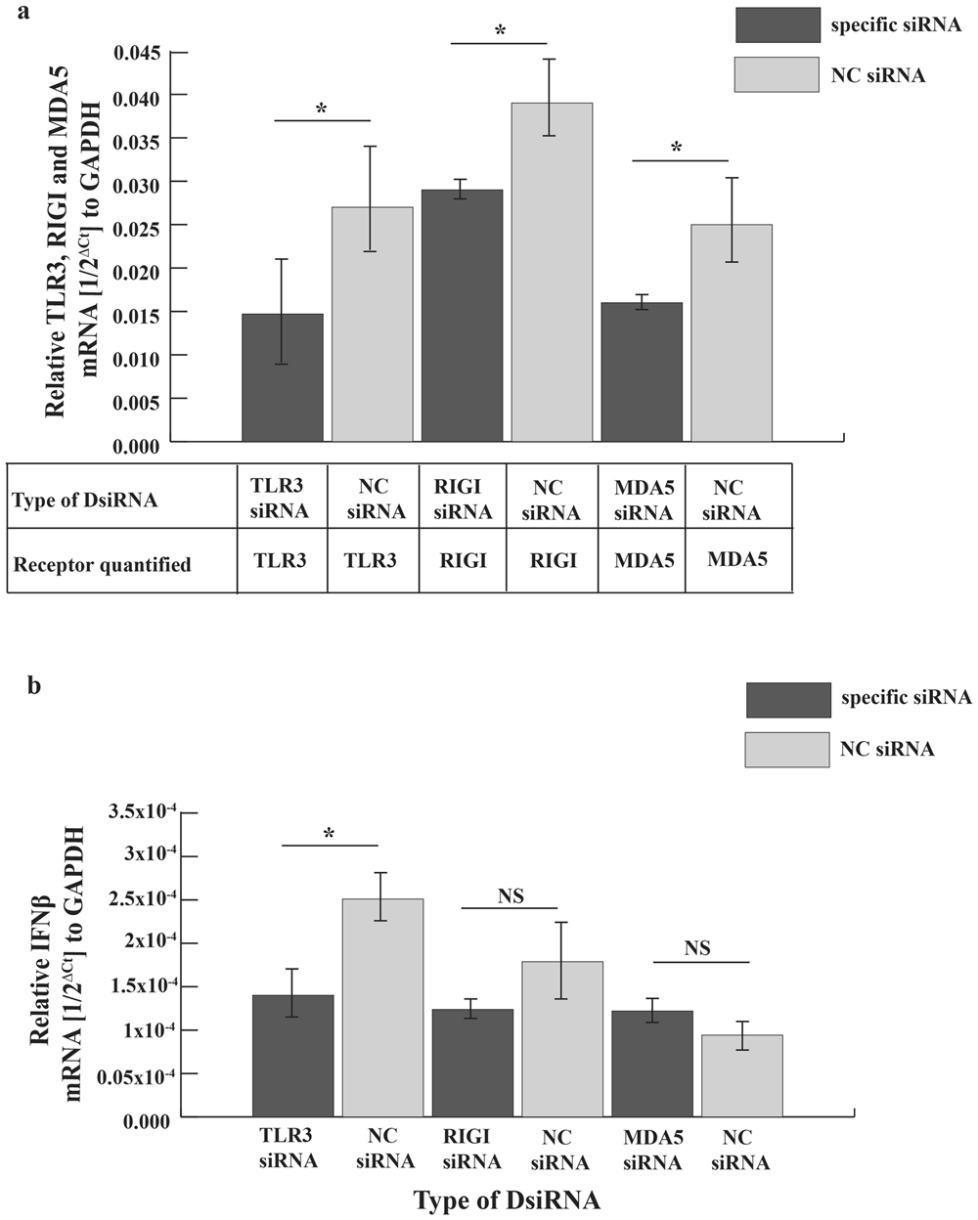
Poly(I:C) signals through TLR3 in Efk3 cells. To identify the role of TLR3, RIGI and MDA5 in poly(I:C) induced interferon signaling, we transfected siRNA specific to *E*. *fuscus* TLR3, RIGI or MDA5 in Efk3 cells and stimulated the cells with poly(I:C). (**a**) siRNA specific to these receptors partially knocked down transcripts for TLR3 (P = 0.043), RIGI (P = 0.021) and MDA5 (P = 0.02) in poly(I:C) stimulated Efk3 cells (mean ± SD, n = 4). (**b**) Knocking down TLR3 in bat cells significantly reduced IFNβ transcripts after treatment with poly(I:C) (mean ± SD, n = 4; P = 0.02). For cells in which RIGI transcripts had been specifically reduced by siRNA, the decrease in IFNβ transcripts was not significant (mean ± SD, n = 4; P = 0.05). MDA5 knockdown did not correlate with decrease in IFNβ transcripts (mean ± SD, n = 4; P = 0.083). Relative amounts of RNA are expressed as a reciprocal of Ct (the PCR cycle at which the product is measurable) normalized to Ct for GAPDH. Statistical significance was calculated using two-tailed Mann-Whitney *U* test for two independent samples. *P < 0.05, NS = not significant. NC = negative control.

To determine if poly(I:C) signaled through an intracellular receptor we measured IFNβ transcripts in bat cells treated either with poly(I:C) added to the medium or poly(I:C) transfected in to bat cells. We compared both treatment types to mock-treated cells. Adding poly(I:C) to the cell culture medium did not increase IFNβ transcripts in bat cells (see Supplemental Fig. S2). To further identify the roles of TLR3, RIGI and MDA5, the three intracellular receptors for dsRNA recognition in bat cells, we partially knocked down these receptors using siRNA. siRNA specific to these receptors significantly reduced transcripts for TLR3, RIGI and MDA5 (Fig. 3a). Knocking down TLR3 transcripts significantly reduced IFNβ transcripts after poly(I:C) transfection (Fig. 3b). Although knocking down RIGI also led to a decrease in IFNβ transcripts post poly(I:C) transfection, it was not significant (Fig. 3b). Knocking down MDA5 did not have any effect on IFNβ transcript levels after poly(I:C) transfection in bat cells (Fig. 3b).

### Poly(I:C) treatment leads to the suppression of the big brown bat wildtype TNFα promoter activity

**Figure 4:**
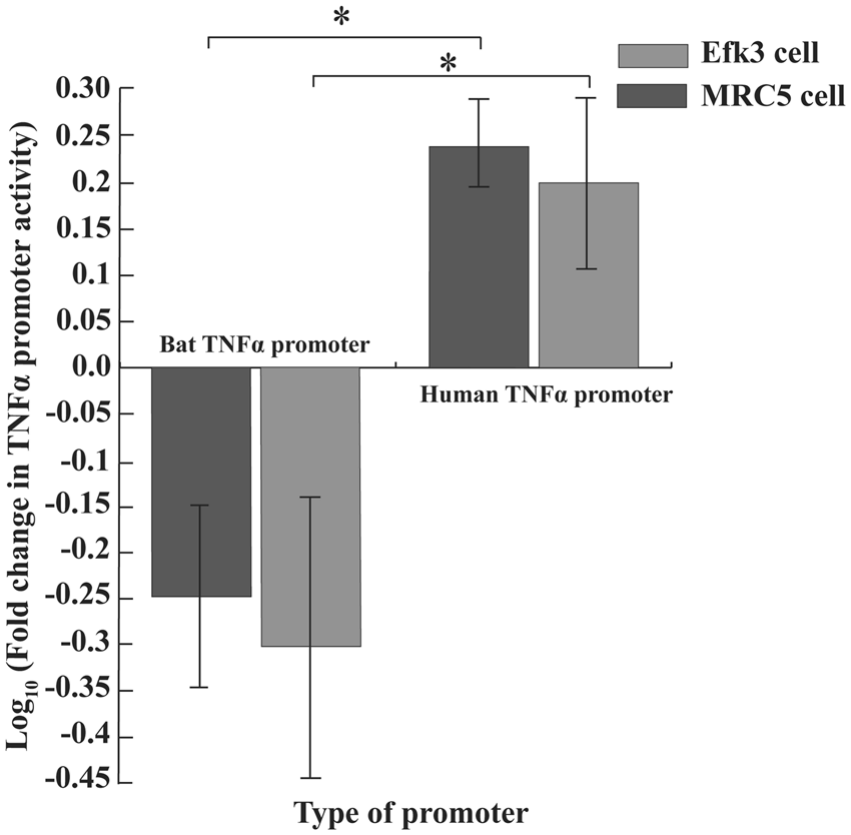
Big brown bat TNFα promoter is functionally different than its human counterpart. We transfected both human and bat TNFα promoters individually in human and bat cells and quantified their activity after poly(I:C) treatment by measuring the expression of a downstream surrogate gene, chloramphenicol acetyltransferase (CAT). Human TNFα promoter showed increased activity after poly(I:C) treatment (as measured by CAT activity) in both Efk3 (P = 0.021) and MRC5 (P = 0.020) cells. In contrast, big brown bat TNFα promoter showed decreased activity after poly(I:C) treatment in both Efk3 (P = 0.021) and MRC5 (P = 0.020) cells (mean ± SD, n = 4). Results are expressed as fold increases over mock-treated cells normalized to β-galactosidase. Statistical significance was calculated using two-tailed Mann-Whitney *U* test for two independent samples. *P < 0.05.

To determine if the difference in the response of EfK3 and MRC5 cells was because of inherent features in their promoters for TNFα, we cloned the human TNFα promoter^38^ and the corresponding region upstream of the big brown bat TNFα coding sequences in a plasmid with the reporter gene, chloramphenicol acetyltransferase (CAT). We transfected the plasmids in MRC5 and Efk3 cells and observed that the big brown bat TNFα promoter showed decreased activity post poly(I:C) challenge in both MRC5 and Efk3 cells (Fig. 4). In contrast, the human TNFα promoter showed increased activity in both Efk3 and MRC5 cells after poly(I:C) challenge (Fig. 4).

### Big brown bat TNFα promoter has a unique c-Rel binding site

**Figure 5:**
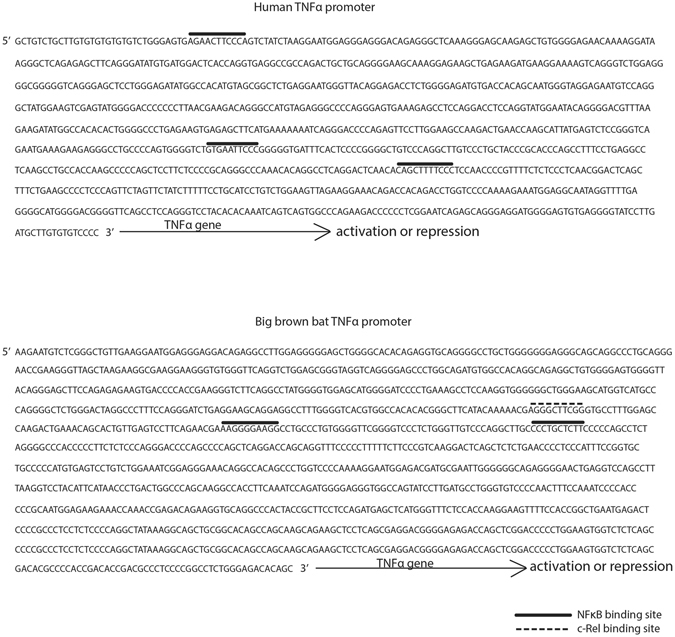
Big brown bat TNFα promoter has a unique c-Rel motif. Human and big brown bat TNFα promoters were analyzed for NFκB and c-Rel transcription factor binding sites. Human TNFα promoter has three NFκB binding and no c-Rel binding site. Big brown bat TNFα promoter has two NFκB binding site and one c-Rel binding site. Other transcription factor binding sites, including sites for NFκB-1 and Rel-A, are not shown here.

Since the big brown bat TNFα promoter showed decreased activity post poly(I:C) stimulation, we examined the human TNFα promoter and bat nucleotide sequence 1,200 bases upstream from the TNFα coding sequence for potential transcription factor binding motifs using the bioinformatics tool PROMO^39^. Both promoters contained motifs for binding NFκB although the big brown bat TNFα promoter had one less site. In addition, the bat promoter had a putative c-Rel binding motif (Fig. 5) not present in the human counterpart.

### c-Rel inhibits big brown bat wildtype TNFα promoter activity

**Figure 6:**
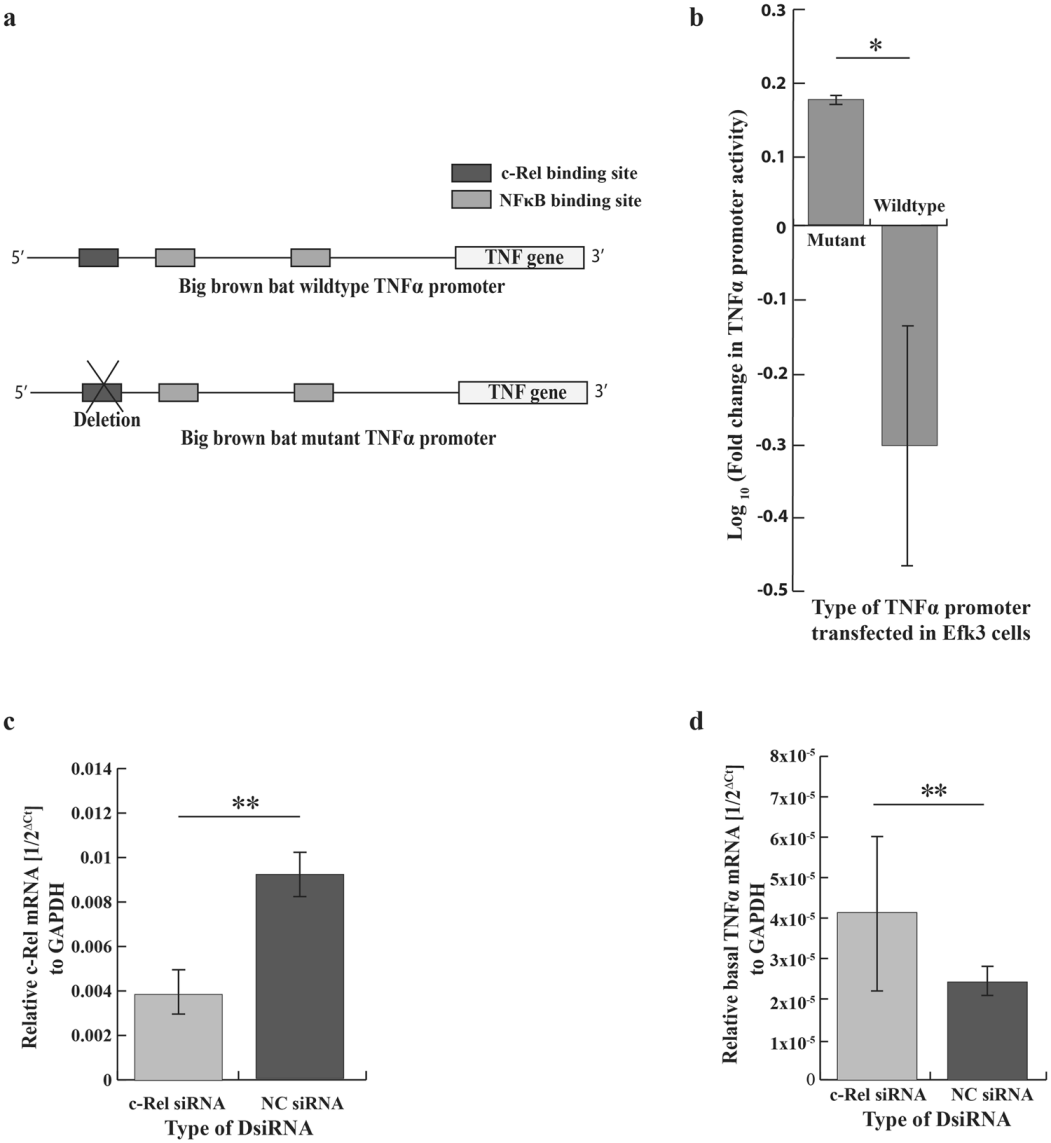
c-Rel acts as a repressor of the big brown bat TNFα promoter. (**a**) Schematic representation of big brown bat TNFα wildtype and mutant promoter. In the mutant bat promoter, the putative c-Rel binding site was deleted. (**b**) Deleting the putative c-Rel binding site in the wildtype big brown bat TNFα promoter significantly (P = 0.034) increased the promoter activity in response to poly(I:C) treatment (mean ± SD, n = 3). (**c**) DsiRNA directed against bat c-Rel significantly (P = 0.009) reduced c-Rel transcripts in bat cells (mean ± SD, n = 5). (**d**) Basal TNFα transcript levels increased significantly (P = 0.009) in partially c-Rel knocked down bat cells (mean ± SD, n = 5). For Fig. 5C and D, relative amounts of RNA are expressed as a reciprocal of Ct (the PCR cycle at which the product is measurable) normalized to Ct for GAPDH. Statistical significance was calculated using two-tailed Mann-Whitney *U* test for two independent samples. *P < 0.05, **P < 0.01. NC = negative control.

By analyzing the nucleotide sequence of the big brown bat TNFα promoter, we identified a potential c-Rel binding site. To identify the role of this binding motif in the big brown bat TNFα promoter, we deleted it (Fig. 6a) and observed the promoter’s activity in response to poly(I:C) in bat cells. Deleting the c-Rel binding site in the big brown bat TNFα promoter increased the promoter activity in response to poly(I:C) (Fig. 6b). To further identify the role of c-Rel in repressing the bat TNFα promoter, we partially knocked down c-Rel transcripts using siRNA in bat cells (Fig. 6c) and quantified basal TNFα transcripts. There was a significant increase in basal TNFα transcripts in these cells (Fig. 6d). We further confirmed that siRNA directed against c-Rel used in this study could successfully shutdown the expression of c-Rel at a protein level (see Supplemental Fig. S3).

### Big brown bat c-Rel responds to poly(I:C) by translocating from the cytoplasm to the nucleus

**Figure 7:**
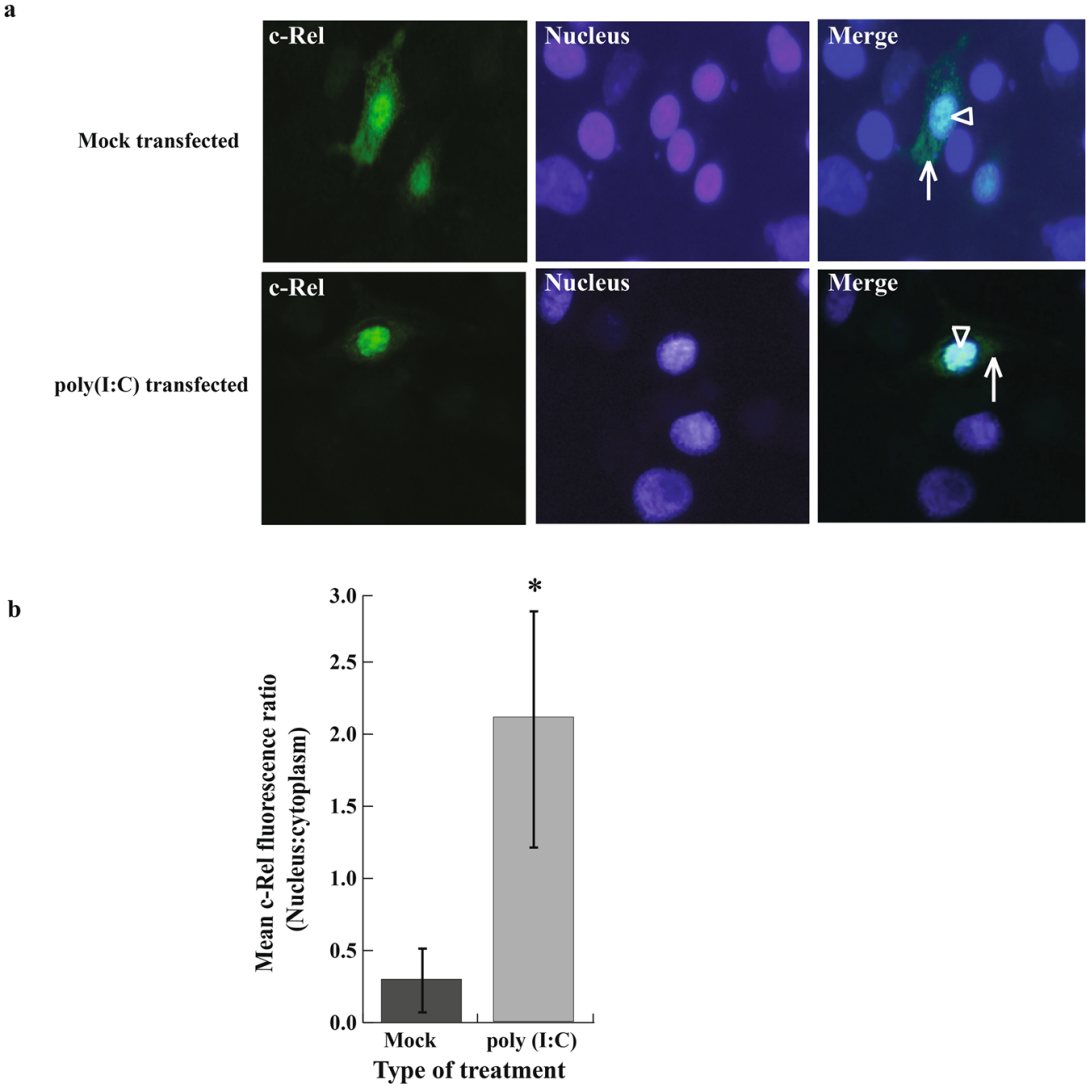
Big brown bat c-Rel localizes in the cell nucleus after poly(I:C) treatment. To characterize the bat c-Rel, we studied the cellular location of ectopically expressed c-Rel in poly(I:C) and mock transfected cells. (**a**) Big brown bat c-Rel localized primarily in the nucleus after poly(I:C) challenge compared to mock challenged cells, where ectopically expressed c-Rel localized both in the cytoplasm and nucleus. (**b**) Mean fluorescence ratio (i.e. nucleus:cytoplasm) was significantly (P = 0.021) higher in poly(I:C) treated cells than mock treated cells (mean ± SD, n = 4). Statistical difference was calculated using two-tailed Mann Whitney *U* test for two independent samples. ^*^ *P* < 0.05.

Rel proteins, either as hetero-dimers or homo-dimers, translocate to the nucleus of the cell after PAMP recognition and downstream signaling by PRRs to bind to promoters and regulate gene transcription. To determine if bat c-Rel responded to poly(I:C) treatment by similar movement, we cloned big brown bat c-Rel into a vector with an influenza virus haemagglutinin (HA) tag, that could be recognized by a commercially available monoclonal antibody, and transfected the protein expressing construct into Efk3 cells. We determined the cellular location of c-Rel by immunofluorescence and observed that c-Rel localized to the nucleus of poly(I:C) treated cells (Fig. 7a). In mock treated cells, c-Rel was present in the nucleus and the cytoplasm (Fig. 7a). The mean nuclear:cytoplasm fluorescence ratio was significantly higher in poly(I:C) treated cells than mock treated cells (Fig. 7b).

### Big brown bat c-Rel binds to the putative c-Rel binding site

**Figure 8:**
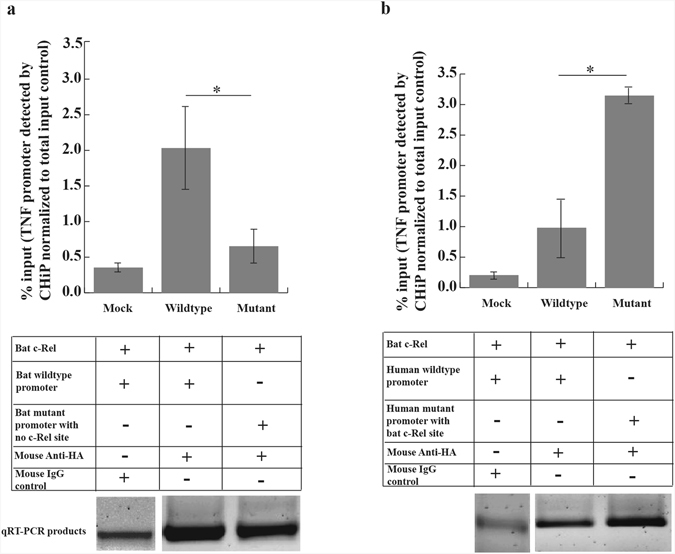
Big brown bat c-Rel binds to the putative c-Rel binding site. We co-transfected plasmids expressing HA-tagged bat c-Rel and human/bat TNFα wildtype/mutant promoters in HEK293T cells. We immunoprecipitated c-Rel using HA-specific antibodies and quantified the amount of TNFα promoter pulled down by qRT-PCR. (**a**) Bat c-Rel pulled down a significantly (P = 0.021) higher amount of wildtype big brown bat TNFα promoter that contained the putative c-Rel binding motif over the mutant promoter in which the motif was deleted (mean ± SD, n = 4). (**b**) Bat c-Rel pulled down a significantly (P = 0.021) higher amount of mutant human TNFα promoter which contained the putative bat c-Rel binding motif over the human wildtype TNFα promoter, which lacked the c-Rel binding motif (mean ± SD, n = 4). qRT-PCR products obtained from quantifying the immunoprecipitated samples were electrophoresed on a gel and their representative cropped images are shown. Full length gel images are shown in Supplemental Fig. S5. Images have been acquired from two different gels. Statistical difference was calculated using two-tailed Mann Whitney *U* test for two independent samples *P < 0.05.

To study if bat c-Rel bound to the putative c-Rel motif, we co-transfected into human cells plasmids expressing HA-tagged bat c-Rel and plasmids containing wildtype or mutant bat and human TNFα promoters. We then performed chromatin immunoprecipitation (ChIP) assay on these cells using antibodies against the HA-tagged c-Rel. We used qRT-PCR to detect TNFα promoters in the immunoprecipitated samples. Bat c-Rel co-precipitated significantly higher amounts of big brown bat wildtype TNFα promoter with the putative c-Rel binding motif than the promoter without the motif (Fig. 8a). In addition, bat c-Rel precipitated a higher amount of the mutant human TNFα promoter with the putative bat c-Rel binding site than the wildtype human promoter (Fig. 8b).

## Discussion

*Chiroptera* is a very diverse order with over 1300 species of bats. Viruses from several families have been detected in different species of bats^4^ although very few of these viruses are known to cause disease in their natural hosts. West Nile virus, Eptesipox virus, a novel group I coronavirus and American bat vesiculovirus have been detected in asymptomatic big brown bats^4, 40^. We do not completely understand how bats and viruses coexist. Researchers are working to identify unique adaptations that might allow bats to coexist with these viruses^5, 41, 42^. Our results indicate that big brown bats may have evolved a unique mechanism to avoid an overblown inflammatory response to activation of the TLR3 pathway by viral ligands. This is in addition to other adaptations in bats currently being proposed, such as loss of the PYHIN family of genes, thereby losing the ability to sense foreign DNA in cells and activating inflammasomes^43^.

To compare the innate responses of human and bat cells to viral ligands we treated the cells with surrogates of viral PAMPs. We used these surrogates instead of viruses capable of infecting both cells to prevent modulation of these pathways by viral proteins. In preliminary experiments we had determined that PED-CoV, which replicates and produces cytopathic effects in bat cells^35^ does not induce an interferon response (data not shown). Coronavirus N protein is known to inhibit IFNβ production by preventing IRF3 phosphorylation^44, 45^.

We observed an increase in IFNβ transcripts in Efk3 cells in response to poly(I:C), as has been previously demonstrated for other bat species^46–48^, but very little increase in TNFα transcripts compared to human cells. Recently, a *P*. *alecto* adaptive immune cell population was characterized and a subset of cells was shown to produce TNFα on stimulation with ionomycin, although the amount of TNFα produced was not reported^49^. We challenged big brown bat bone marrow derived myeloid cells and the kidney cell line with poly(I:C) and quantified the transcripts for representative antiviral and inflammatory genes. Inflammatory cytokine transcripts for TNFα, IL8 and IL1β (see Supplementary Table S2) in Efk3 cells and TNFα in big brown bat bone marrow derived myeloid cells were not expressed to levels observed in MRC5 cells. IFNβ and TNFα transcript levels observed in poly(I:C) challenged big brown bat myeloid cells were comparable to levels observed in poly(I:C) challenged *P*. *alecto* bone marrow derived dendritic cells by Zhou *et al*.^50^.

Interferon β production in response to poly(I:C) has been studied in bat cells^35, 47^ but the receptors involved in double-stranded RNA signaling have not been fully explored. Adding poly(I:C) to the culture medium did not upregulate transcripts for IFNβ in bat cells (see Supplemental Fig. S2) suggesting that the receptor recognizing poly(I:C) was intracellular. The requirement of transfection for the activation of TLR3 in certain cell types is supported by Zhou *et al*.^51^. Using siRNA, we were able to show for the first time that poly(I:C), and likely dsRNA, is recognized primarily through TLR3 in bat cells (Fig. 3). However, according to our results the role of RIGI in dsRNA recognition cannot be completely ruled out. The role of other PRRs in bats in recognizing specific ligands is yet to be explored.

TNFα plays a key role in inflammatory, infectious and malignant conditions. TNFα signaling transduction pathways are complex and are not fully understood^52^. NFκB plays a central role in the regulation of TNFα gene expression. Different combinations of the subunits that make up NFκB have vastly different effects on gene expression^18^. For instance, hetero-dimers of p50 or p52 and p65 or RelB activate transcription. In contrast, c-Rel as a homo-dimer or in association with p50 and p65, repress transcriptional activation by NFκB^19^ and c-Rel has been previously shown to be a repressor of certain gene promoters in human cells, such as Ephrin type-B receptor 2 (EPHB2) in colorectal cancer cells^36^. We detected a putative c-Rel binding motif in the big brown bat TNFα promoter. Deleting this motif reversed the bat promoter activity after poly(I:C) treatment and partial knockdown of c-Rel RNA significantly increased basal TNFα transcript levels in bat cells demonstrating the ability of c-Rel to repress the TNFα promoter in bat cells.

The big brown bat TNFα promoter had two NFκB binding sites, which is one less than the human counterpart. We do not know if having one less NFκB binding site can be an additional reason for the low TNFα promoter activity. However, adding an additional NFκB binding site to the big brown bat promoter lacking the c-Rel binding site did not cause any further increase in promoter activity after poly(I:C) treatment (data not shown).

c-Rel might not be the only regulator of inflammatory gene expression and big brown bats may have evolved more than one mechanism to regulate inflammatory responses. We evaluated a second inflammatory gene, IL1β and found that it, like TNFα, did not respond to poly(I:C) in bat cells (see Supplementary Table S2). However, while the IL1β promoter had identifiable NFκB binding sites, it did not have a specific c-Rel binding site (data not shown). There is evidence that NFκB molecules comprising p50 homodimers can act as transcriptional repressors (reviewed by Rothwarf and Karin^53^). Further work is required to identify the role of p50 homodimers in bat cells.

Zhang *et al*. have reported *c-Rel* to be under positive selection in bats based on whole-genome analysis of two distantly related species, fruit bat *P*. *alecto* and insectivorous bat *Myotis davidii* ^54^. They suggest that the selection may have been driven by the involvement of c-Rel in DNA repair pathways and the need for efficient repair of damage caused by reactive oxygen species generated during flight. In addition, Enchéry and Horvat have also speculated that the positive selection of *c-Rel* may contribute to bats’ immunovirological peculiarities^55^. Our results suggest that the positive selection of *c-Rel* may also have been driven by the need to dampen the destructive effects of inflammation in response to viral infections. Zhang *et al*. also identified mutations in the REL homology domain (RHD) of c-Rel that could potentially affect the binding of IκB and speculated that this may allow nuclear transport in the absence of TLR3 stimulation^54^. These mutations are also present in big brown bat *c-Rel* (see Supplementary Fig. S4). However, we did find that while ectopically expressed bat c-Rel was present in both cytoplasm and nucleus, poly(I:C) stimulation increased translocation to the nucleus (Fig. 7a). The mutations, therefore, do not completely obviate TLR3 control.

We have also shown that big brown bat c-Rel physically interacts with the putative c-Rel binding site. Promoters containing the putative c-Rel binding site were co-immunoprecipitated at higher levels by the bat c-Rel than promoters that lacked it (Fig. 8). These results demonstrate that bat c-Rel can suppress the expression of TNFα and that its putative binding site in the promoter for the gene plays a role. However, we have not identified the mechanism by which c-Rel acts. More work is also needed to characterize the interaction between the various NFκB subunits and their downstream effects on different promoters.

**Figure 9:**
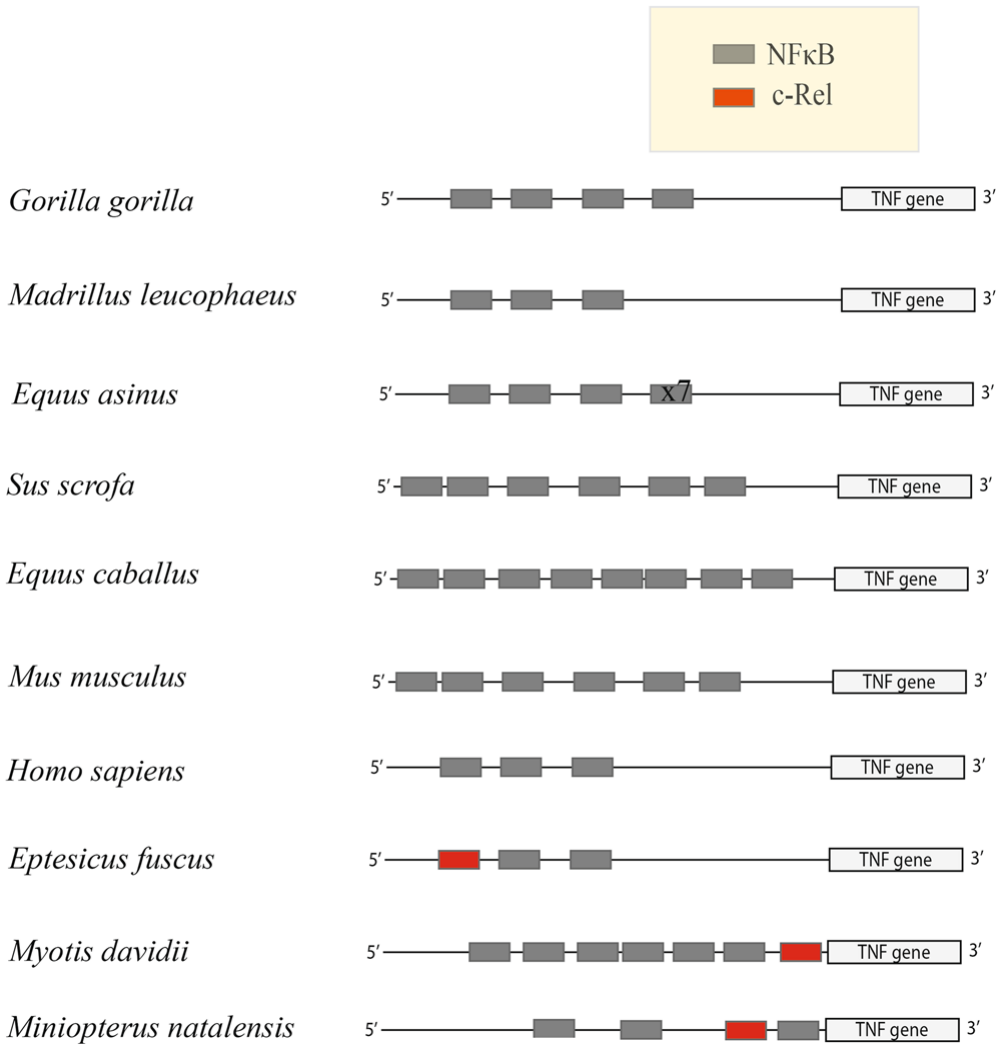
Bats are unique in having c-Rel binding sites in their TNFα promoter. At least three species of bats, *E*. *fuscus*, *M*. *davidii* and *M*. *natalensis* have c-Rel binding sites upstream of the TNFα gene. c-Rel sites (red boxes) are absent in the 1000 bp region upstream of the TNFα gene in other mammals represented in this figure. The number of NFκB binding motifs (gray boxes) in the TNFα promoter varied amongst mammals. All sites were predicted using PROMO. For *Equus asinus* x7 indicates 7 additional copies of the NFκB binding site.

Proteins of the Rel family differ according to tissue types in humans. Rel family protein, p65 is found in virtually all cell types, whereas c-Rel complexes (eg. p50/c-Rel and c-Rel homo-dimers) are predominantly expressed in cells of hematopoietic lineage, such as lymphoid and myeloid cells^37^. We detected c-Rel transcripts in a wide variety of big brown bat tissues such as spleen, gut, ileum, kidney, lung, liver and the kidney cell line (Efk3) as well (see Supplementary Table S3). We further analysed the promoters of animals in different mammalian orders and could not detect a c-Rel binding motif in the sequence 1000 bp upstream of their TNFα genes. We detected potential c-Rel binding sites in other bats such as *M*. *davidii* and *M*. *natalensis* (Fig. 9). We did not detect a potential c-Rel binding site in the *P*. *alecto* DNA sequences that lie upstream from the TNFα coding sequences but there was one downstream of the coding sequences (data not shown). The potential role of c-Rel in the DNA repair pathway and evolution of flight in bats has been proposed by Zhang *et al*.^54^. We do not yet fully understand the role of c-Rel in DNA repair pathways in different tissue types in bats and in different species of bats. Different bat species could have evolved different strategies or a combination of strategies to control an overblown inflammatory response.

Our study demonstrates that big brown bats have possibly evolved a mechanism to control the over expression of inflammatory genes in response to activation of their innate immune system by viral nucleic acid PAMPS. Our work raises several questions about the bat innate immune response that need to be further explored. Identifying unique defence mechanisms in bats might allow us to extend the knowledge for therapeutic purposes in spill-over hosts that often develop significant disease or succumb to infections with these viruses.

## Materials and Methods

### Ethics statement

Long bones (femur and humerus) and organs were obtained from big brown bats submitted to Canadian Wildlife Health Cooperative (CWHC). The bats were euthanized by a protocol approved by the Committee on Animal Care and Supply of the University of Saskatchewan Animal Research Ethics Board (protocol #20090036) and were in accordance with regulations approved by the Canadian Council on Animal Care.

### Cell culture

*Eptesicus fuscus* kidney cells (Efk3) were grown in Dulbecco’s Minimal Essential Medium with GlutaGro (DMEM; Corning) containing 10% fetal bovine serum (FBS; Seradigm), Penicillin/Streptomycin (Gibco) and 1% GlutaMax (Gibco). MRC5 cells (ATCC CCL-171) were cultured in Minimum Essential Medium Eagle (MEM; Corning) supplemented with 10% FBS, 1/100 non-essential amino acids (NEAA; Gibco), 1/100 4-(2-hydroxyethyl)-1-piperazineethanesulfonic acid (HEPES; Gibco) and 1/1000 gentamycin (Gibco). HEK293T cells (Dr. Robert Brownlie, VIDO-Intervac) were cultured in DMEM with GlutaGro containing 10% FBS and Penicillin/Streptomycin. For bone marrow derived myeloid cells, bone marrow from big brown bat long bones was processed as described for mice^56^. The cells were seeded in Roswell Park Memorial Institute 1640 (RPMI; Sigma-Aldrich) medium containing 10% FBS, Penicillin/Streptomycin and 20 ng/ml human granulocyte-macrophage colony-stimulating factor (hGM-CSF; PeproTech).

### TLR challenge

MRC5, Efk3 and big brown bat bone marrow derived myeloid cells were seeded at a concentration of 3 × 10^5^ cells/well in 6 well plates and transfected with TLR ligands. Briefly, cell lines were transfected with 750 ng/ml poly(I:C) (InvivoGen) or 4 μg/ml single-stranded RNA 40 (ssRNA40; InvivoGen) or 3 μM CpG ODN (InvivoGen) using Lipofectamine 2000 (Invitrogen). Cells were harvested 16 h post-transfection and RNA was extracted. For time-point experiments, cell lines were treated with the above-mentioned concentrations of TLR ligands and RNA was extracted at indicated time points.

### Nucleic acid extraction, PCR and qRT-PCR

All RNA extractions were performed using the RNeasy Plus Mini kit (QIAGEN, Germany) as per manufacturer’s instructions. cDNA was prepared using the QuantiTect Reverse Transcription kit (QIAGEN) as per manufacturer’s instructions. One μg of RNA was used for cDNA preparation. cDNA was used as a template for the quantification of target genes. DNA extraction from MRC5 and Efk3 cells was performed using the DNeasy Blood and Tissue kit (QIAGEN) as per manufacturer’s instructions.

Conventional PCR was performed to amplify human or big brown bat TNFα promoters, big brown bat c-Rel coding sequence (CDS) and cDNA from c-Rel transcripts in big brown bat organs using specific primers. Primers with restriction sites were used to clone the TNFα promoters and c-Rel CDS (Table 1). Primers without restriction sites were designed to detect c-Rel transcripts in big brown bat organs (see Supplementary Table S4). Human TNFα promoter sequence was obtained from NCBI (Accession number: AB048818) and amplified by PCR from DNA extracted from MRC5 cells. The big brown bat TNFα promoter was defined as a sequence up to 1200 bp upstream of the TNFα gene (sequence submitted; GenBank accession: BK009991) and amplified by PCR from DNA extracted from Efk3 cells. Big brown bat c-Rel sequence was obtained from NCBI (Accession number: XM_008162099.1). PCR was performed using the following thermal cycle profile: initial denaturation for 3 min at 94 °C, 35 PCR cycles at 94 °C/30 s, 55 °C/30 s and 72 °C/1 min. The final extension was at 72 °C for 10 min.

**Table 1:** siRNA, cloning and ChIP qRT-PCR primer sequences.

Sequence	Primers	Features and legend
Human TNF alpha promoter	GCCGGTACCGCTGTCTGCTTGTGTGTGTG and GCCCTCGAGGGGGACACACAAGCATCAAG	KpnI and XhoI sites
Big brown bat TNF alpha promoter	GCCACGCGTAAGAATGTCTCGGGCTGTT and GCCCTCGAGGCTGTGTCTCCCAGAGGCC	MluI and XhoI sites
Big brown bat cRel CDS cloned in pCMV-HA-N	GCCGTCGACCATGCGTTTTCGATACAAATG and GCCGCGGCCGCTTACAAGTTAACCGGAAAAA	SalI and NotI sites
CD.Ri.17417.13.1 (cRel_siRNA) - sequence	rArArA rGrGrA rArGrC rUrArU rUrArU rUrUrC rArArG rArATA	r = ribose sugar
CD.Ri.17417.13.1 (cRel_siRNA) - sequence2	rUrArU rUrCrU rUrGrA rArArU rArArU rArGrC rUrUrC rCrUrU rUrArC	r = ribose sugar
CD.Ri.17417.13.2 (cRel_siRNA) - sequence	rGrGrA rArGrA rUrUrC rArUrU rArArA rArArA rGrArA rUrCA A	r = ribose sugar
CD.Ri.17417.13.2 (cRel_siRNA) - sequence2	rUrUrG rArUrU rCrUrU rUrUrU rUrArA rUrGrA rArUrC rUrUrC rCrUrU	r = ribose sugar
Big brown bat TNF alpha promoter - ChIP primers	GGCAGATGTGGCCACAGGCAGAG and CAGAGAGCTGAGTCCTTGACG	—
Human TNF alpha promoter - ChIP primers	GGGGAGAACAAAAGGATAAGG and CTCTCACTTCTCAGGGCCCCAG	—

For siRNA sequences, ribonucleotides are preceeded by the letter ‘r’. Cloning primer sequences contain restriction sites as part of the sequence.

For the quantification of innate immune response genes, qRT-PCR assays targeting respective gene transcripts (see Supplementary Table S4) and the normalizer (Glyceraldehyde-3-phosphate; GAPDH) were performed for both MRC5 and Efk3 cells. Agilent’s MX3005P PCR cycler was used in conjunction with Quantifast SYBR Green PCR kit (QIAGEN) and samples were prepared as previously mentioned^57^. Primers for Efk3 cells were designed using the annotated big brown bat genome (Accession No. PRJNA72449). Primer sequences for MRC5 cells were obtained from PrimerBank^58, 59^ or nucleotide database on National Centre for Biotechnology Information (NCBI). When primer sequences were not available for MRC5 or genes not annotated for big brown bat, multiple sequence alignment was performed with other mammalian homologues and primers were designed against conserved regions. One of the cytokines, IL8, is not annotated in the big brown bat genome. Primers for IL8 were designed using the annotated *Myotis lucifugus* genome. The products were quantified based on the amount of relative gene expression. All amplified products were confirmed on a gel and sequenced (Macrogen). Reaction efficiencies for qRT-PCR primers were between 95 and 105%.

For qRT-PCR, after the initial denaturation step of 95 °C for 5 minutes, two step cycling for 40 cycles was performed at 95 °C/10 s, (51–56) °C/30 s. Absorbance readings were acquired after each cycle. The final three steps were carried out at 95 °C/1 min, 55 °C/30 s and 95 °C/30 s to generate the dissociation curve. Absorbance readings for the dissociation curve were acquired at every degree from 55–95 °C. The annealing temperatures were optimized for different groups of genes (see Supplementary Table S4). Relative fold change in gene expression between the two groups of cells (treated and mock treated) was calculated after normalizing the Ct values using GAPDH. Three housekeeping genes were tested (GAPDH, β-actin and β-2-microglobulin) for MRC5 and two for Efk3 cells (GAPDH and β-actin). There was no variation in Ct values for the housekeeping genes between treated and mock treated samples. Thus GAPDH was chosen for normalizing the data. Difference of one Ct indicates a two-fold difference in gene expression.

qRT-PCR for quantifying immunoprecipitated DNA after the ChIP assay was performed using primers (Table 1) designed to amplify a region spanning the putative c-Rel binding site of approximately 480 bp for the bat promoter and 410 bp for the human promoter (±the putative c-Rel binding motif). The reaction conditions were as described above.

### Agarose Gel Electrophoresis

One percent agarose (Invitrogen, USA) gels were prepared using 0.5X TBE [Tris – 1 M (VWR), Ethylenediaminetetraacetic acid disodium salt (EDTA) solution – 0.02 M (Gibco) and Boric acid – 1 M; pH 8.4]. One ul SYBR Safe DNA gel stain (Invitrogen) was added for every 1 ml of gel. Ten μl of PCR or qRTPCR products were run on the gel for 1 h at 105 volts and visualized under an ultraviolet gel imaging system (AlphaImager HP).

### Cloning TNFα promoter and c-Rel

Human TNFα promoter sequence was amplified by PCR from DNA extracted from MRC5 cells and cloned in pCAT3 vector (Promega) upstream of the chloramphenicol acetyltransferase (CAT) gene using restriction sites KpnI and XhoI. The big brown bat TNFα promoter was amplified by PCR using DNA extracted from Efk3 cells and cloned upstream of the CAT gene in a pCAT3 vector using restriction sites MluI and XhoI. Big brown bat c-Rel coding sequence (CDS) was amplified from cDNA prepared from RNA extracted from Efk3 cells and cloned in-phase downstream of a Hemagglutinin (HA) tag in pCMV-HA-N vector (Clontech) using restriction sites SalI and NotI.

### Generating TNFα promoter mutants

Mutant big brown bat and human TNFα promoters were generated by removing or adding the c-Rel binding site. Agilent’s QuikChange II Site-Directed Mutagenesis kit was used as per a modification of the manufacturer’s protocol suggested by Wang and Malcolm^60^. For the bat mutant promoter, two primers (IDT) were designed to loop out the c-Rel binding site, BBB M1 – F - GCTTCATACAAAAACTGCCTTTGGATCCAAG and BBB M1 – R – CTTGGATCCAAAGGCAGTTTTTGTATGAAGC. The primers were used to amplify wild-type big brown bat TNFα promoter. For the human mutant promoter, primers were designed containing the putative bat c-Rel binding motif: Hu-M1-F- GAATGGGTTACAGGAGGGGCTTCGGATCCTCTGGGGAGATG and Hu-M1-R- CATCTCCCCAGAGGATCCGAAGCCCCTCCTGTAACCCATTC. The primers were used to amplify wild-type human TNFα promoter. Deletion and addition of the c-Rel binding site were confirmed by sequencing (Macrogen).

### Chloramphenicol acetyl transferase (CAT) and β-galactosidase (β-gal) assay

MRC5 and Efk3 cells were seeded at a concentration of 3 × 10^5^ cells/well in 6 well plates. At 60–80% confluency, 500 ng of human or big brown bat TNFα promoter (wildtype or mutant), 500 ng β-galactosidase (β-gal) expressing plasmid and 1 μg of pcDNA empty plasmid to make up a total of 2 μg DNA/well was transfected using Lipofectamine 2000. After 24 h, the medium was replaced with fresh complete medium (DMEM). After 4 h, cell lines were transfected with 750 ng/ml poly(I:C) and incubated for 16 h. CAT and β-gal assays were performed as previously mentioned^61^.

### Partial knock-down of c-Rel, TLR3, RIGI and MDA5 transcripts in Efk3 cells

Dicer-ready siRNA (DsiRNA) specific to big brown bat c-Rel, TLR3, RIGI and MDA5 were designed and obtained through Integrated DNA Technologies (IDT). A 100 nM final concentration of a 1:1 mixture of two DsiRNAs (Table 1 and see Supplementary Table S5) targeting separate regions on the respective transcript was transfected into Efk3 cells using Lipofectamine 2000. Scrambled non-specific DsiRNA (NC DsiRNA; IDT) was used as a negative control. Cy3 labelled DsiRNA (IDT) was used to confirm 100% transfection efficiency.

### Immunofluorescence

Efk3 cells were seeded at a concentration of 3 × 10^5^ cells/well in 6 well plates with glass cover-slips and transfected with 5 μg/well pCMV-HA-N plasmid expressing big brown bat c-Rel using Lipofectamine 2000. Cells were treated with 750 ng/ml poly(I:C) after 24 h and incubated for another 16 h. Media was discarded and cells were rinsed with 2 ml PBS. Cover-slips were transferred to wells containing ice-cold methanol in 6-well plates and incubated for 20 mins in a freezer. Methanol was discarded and cells were washed with PBS. Cells were blocked using a blocking solution [PBS, 10% newborn calf serum (Invitrogen) and 0.1% Tween 20 (USB)]. Primary staining for c-Rel was performed using 1:2000 dilution (as used by Smith *et al*.^62^) of mouse anti-HA (Sigma). Secondary staining was performed using 4 μg/ml goat anti-mouse Alexa 488 (Molecular Probes) and 0.2 μg/ml Hoechst 33342 (Molecular Probes) in blocking solution. Cells were observed under a fluorescent microscope and images were acquired using DP Controller (OLYMPUS, Version 3.2.1.276). Mean fluorescence was measured using Image J (Version 1.49) and calculated using a formula previously described^63^.

### Differential staining of bone marrow derived cells

Cells obtained from big brown bats bone marrow were concentrated onto a slide using Cytospin 4 (ThermoFisher). The slides were fixed in Hema 3 fixative solution (Fisher Scientific, USA) for 10 seconds, followed by 5 dips for 1 second each in Hema 3 solution I (Fisher Scientific, USA) then Hema 3 solution II (Fisher Scientific, USA). The slides were rinsed with deionized water, air dried and observed under a light microscope.

### Chromatin immunoprecipitation assay (ChIP)

HEK293T cells were seeded in 6-well plates at a concentration of 3 × 10^5^ cells/well. Cells at 60–70% confluency were co-transfected with the TNFα promoters and big brown bat c-Rel using Lipofectamine 2000 in serum free medium (OPTI-MEM, Gibco). After 4 h, serum free medium was replaced with complete medium and the cells were incubated for 16 h. After 16 h, cells were transfected with 750 ng/ml poly(I:C) and incubated for 4 h. The cells were then fixed using 1% formaldehyde (Thermo Scientific) and processed for ChIP assay as per manufacturer’s instructions (Pierce Agarose ChIP kit, Thermo Scientific). For immunoprecipitating HA-tagged big brown bat c-Rel, 1/1000 dilution of mouse anti-HA antibody (Sigma) was used and 5μg mouse IgG isotype control (Thermo Scientific) was used as the non-specific antibody control. ChIP assay positive (human anti-RNA polymerase II) and negative control (rabbit IgG) antibodies were provided with the kit. Positive control primers for human GAPDH were provided with the kit. The amount of TNFα promoter immunoprecipitated was quantified by quantitative real-time PCR (qRT-PCR) and percent input was calculated and plotted as per manufacturer’s instructions and as previously mentioned^64^. The qRT-PCR products were analysed by gel electrophoresis.

### Statistics

Significance of the data was determined by two-tailed Mann Whitney *U* test for non-parametric independent samples using IBM SPSS (Version 21). In the figures, *P < 0.05 and **P < 0.01. Actual ‘P values’ are mentioned in figure legends.

## Electronic supplementary material

Supplementary information
