# Supplementary material for: Lack of inflammatory gene expression in bats: a unique role for a transcription repressor
Source: Sci Rep. 2017 May 22;7:2232. doi: 10.1038/s41598-017-01513-w (PMC5440382; doi:10.1038/s41598-017-01513-w)
Supplement: Supplementary file 1 — Supplementary information [file 41598_2017_1513_MOESM1_ESM.pdf]

Supplemental Information

Title: Lack of inflammatory gene expression in bats: a unique role for a transcription repressor.

Authors: Arinjay Banerjee[a], Noreen Rapin[a], Trent Bollinger[b] and Vikram Misra[a]#

Affiliations: [a] Department of Microbiology, Western College of Veterinary Medicine, University of Saskatchewan, Saskatoon, Saskatchewan, Canada, S7N 5B4.

[b] Department of Pathology, Western College of Veterinary Medicine, University of Saskatchewan, Saskatoon, Saskatchewan, Canada, S7N 5B4.

Supplemental Figure S1

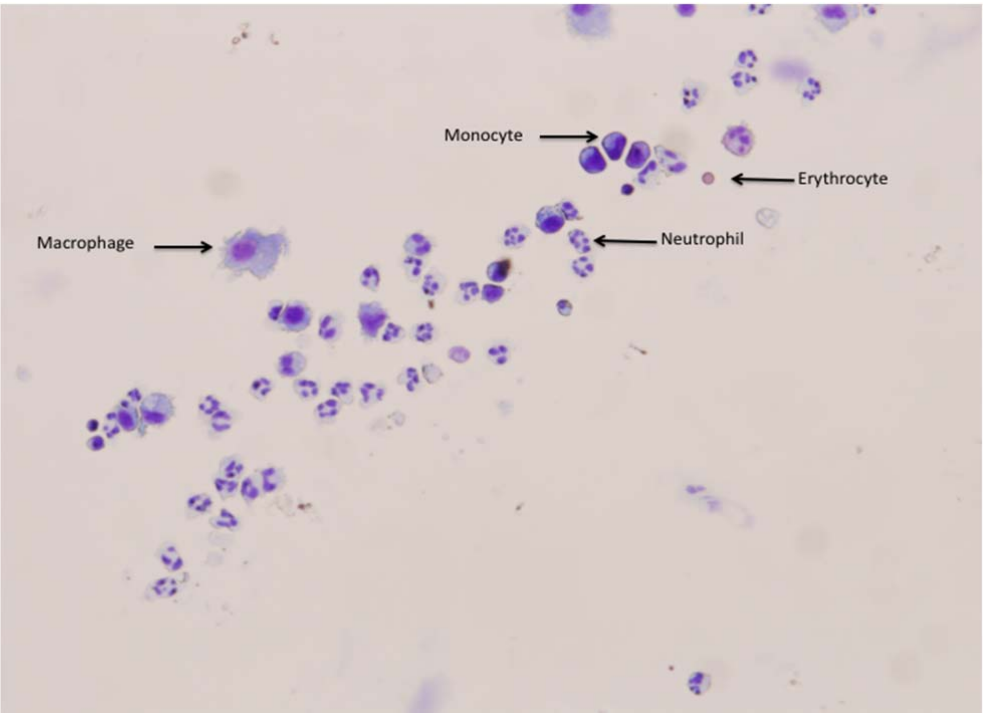

Figure S1. Big brown bat bone marrow derived myeloid cells were a mixed population. Bone marrow derived cells were deposited on a slide by centrifugation and differentially stained. Various populations of myeloid cells were observed. Cell types based on morphological features are labelled in the figure.

Supplemental Figure S2

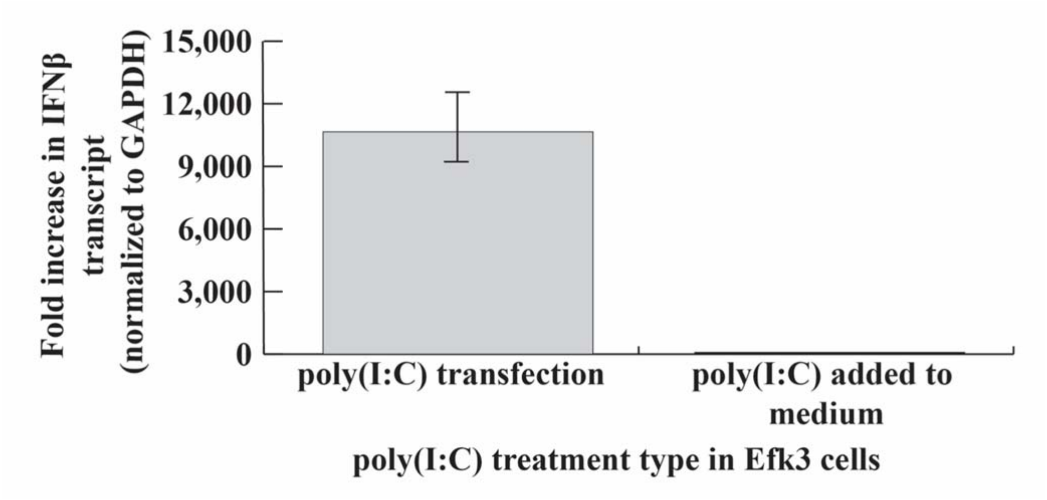

Figure S2. Transfection of poly(I:C) is essential to activation of the TLR3 pathway. To determine if poly(I:C) is recognized by a cell surface receptor or an internal receptor, such as TLR3, in bat cells, we quantified IFNβ transcripts in Efk3 cells after transfecting or adding poly(I:C) to culture medium. Adding poly(I:C) to the culture medium did not upregulate IFNβ transcripts in Efk3 cells, whereas transfecting poly(I:C) induced several thousand folds of IFNβ transcripts (Mean±SD, n=4).

Supplemental Figure S3

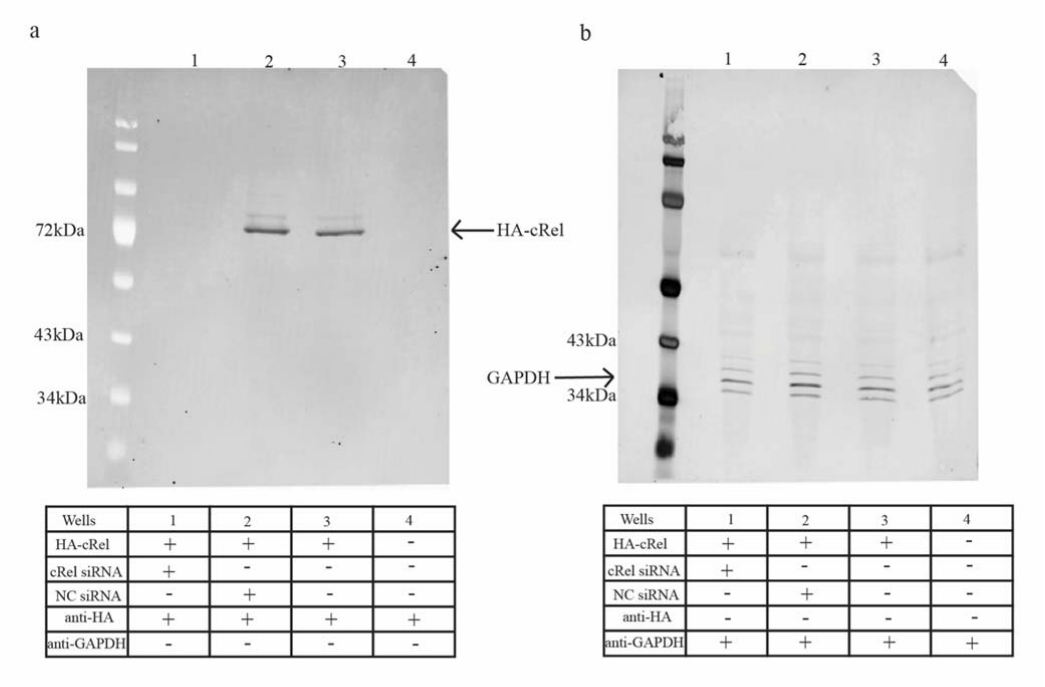

Figure S3. c-Rel siRNA shuts down expression of HA-tagged c-Rel. To study the knockdown potential of c-Rel siRNA at a protein level, we simultaneously transfected HEK293T cells with siRNA specific to c-Rel, non-specific scrambled siRNA (NC siRNA) or no siRNA and plasmids expressing HA-tagged c-Rel. (a) siRNA specific to c-Rel completely shuts down the expression of HA-c-Rel from the plasmid. In contrast, cells transfected with NC siRNA or no siRNA express HA-c-Rel. (b) There was no change in GAPDH expression between different treatments. NC = Negative control.

Supplemental Figure S4

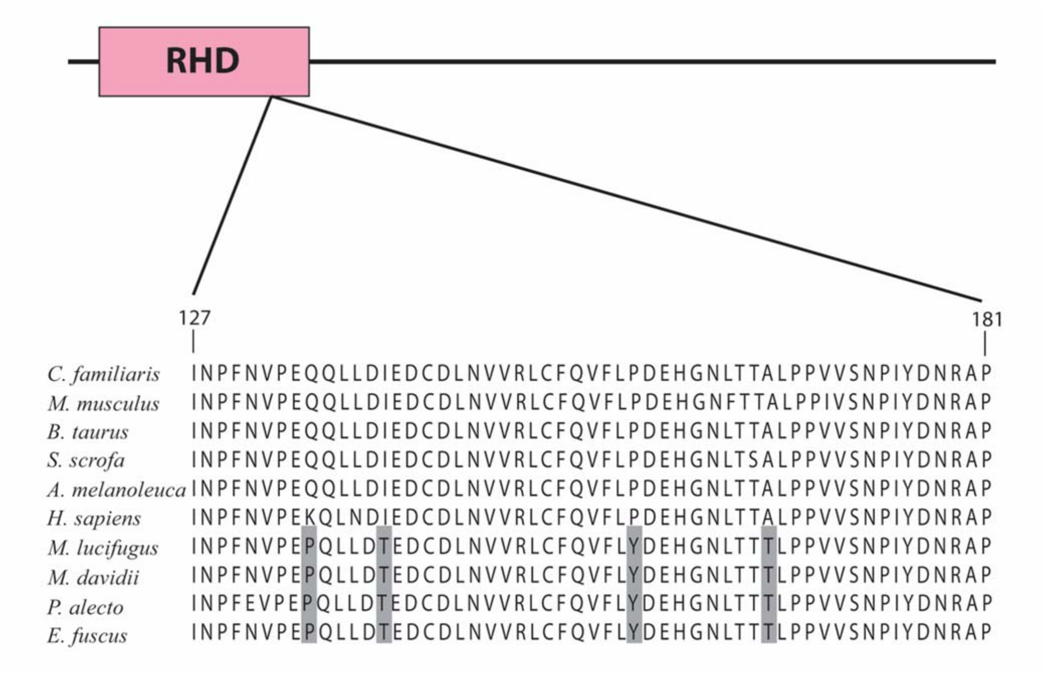

Figure S4. Mutations in the c-Rel Rel homology domain (RHD). Zhang *et al.* show that 4 mutations specific to bat c-Rel exist in the downstream region of the RHD [1]. IκB binding domain is located in the downstream region of the RHD and Zhang *et al.* propose mutations in this region can affect IκB binding. Differences in amino acids between bat RHD domains and those of other representative mammals are highlighted.

Supplemental Figure S5

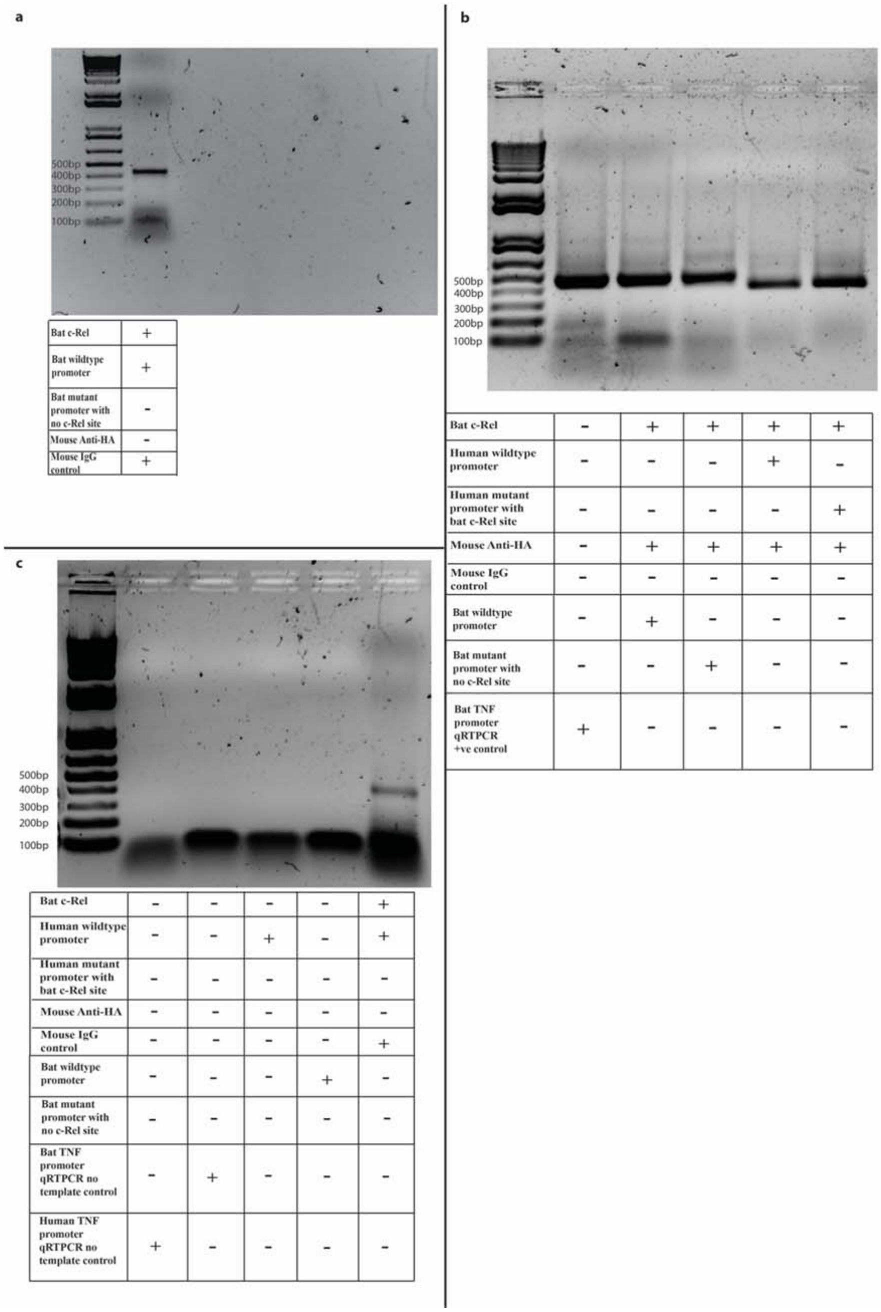

a

Bat c-Rel	+
Bat wildtype promoter	+
Bat mutant promoter with no c-Rel site	-
Mouse Anti-HA	-
Mouse IgG control	+

b

Bat c-Rel	-	+	+	+	+
Human wildtype promoter	-	-	-	+	-
Human mutant promoter with bat c-Rel site	-	-	-	-	+
Mouse Anti-HA	-	+	+	+	+
Mouse IgG control	-	-	-	-	-
Bat wildtype promoter	-	+	-	-	-
Bat mutant promoter with no c-Rel site	-	-	+	-	-
Bat TNF promoter qRTPCR +ve control	+	-	-	-	-

c

Bat c-Rel	-	-	-	-	+
Human wildtype promoter	-	-	+	-	+
Human mutant promoter with bat c-Rel site	-	-	-	-	-
Mouse Anti-HA	-	-	-	-	-
Mouse IgG control	-	-	-	-	+
Bat wildtype promoter	-	-	-	+	-
Bat mutant promoter with no c-Rel site	-	-	-	-	-
Bat TNF promoter qRTPCR no template control	-	+	-	-	-
Human TNF promoter qRTPCR no template control	+	-	-	-	-

Figure S5. qRT-PCR products from ChIP assay were electrophoresed on separate gels. Images for figure 8 in the article file are shown above. Cropped images

were acquired from these images and scaled to fit under the qRT-PCR data in Fig. 8. ChIP and qRT-PCR controls are shown too.

Supplemental Table S1. PRR transcripts detected in human (MRC5) and bat (Efk3) cells by PCR.

PRR type	MRC5	Efk3
**TLR2**	+	+
**TLR3**	+	+
**TLR7**	+	+
**TLR8**	+	+
**TLR9**	+	+
**RIG-I**	+	+
**MDA5**	+	+

Supplemental Table S2. Fold changes observed for innate immune gene transcripts in MRC5 and Efk3 cells after different TLR ligand treatments. We transfected human fibroblasts and bat kidney (Efk3) cells with poly(I:C), a known TLR3 stimulant, and studied the expression of genes involved in the TLR3 pathway, relative to mock transfected cells. Experiments were carried out in replicates (n=3). Standard deviation values are reported along with mean fold changes.

**TL R liga**	**poly (I:C)**				**ssRN A 40**				**CpG ODN**			

**nd**												
**Gene**	**Mean fold upregulation - MRC5 cells**	**Standard deviation - MRC5 cells**	**Mean fold upregulation - Efk3 cells**	**Standard deviation - Efk3 cells**	**Mean fold upregulation - MRC5 cells**	**Standard deviation - MRC5 cells**	**Mean fold upregulation - Efk3 cells**	**Standard deviation - Efk3 cells**	**Mean fold upregulation - MRC5 cells**	**Standard deviation - MRC5 cells**	**Mean fold upregulation - Efk3 cells**	**Standard deviation - Efk3 cells**
**IRF3**	1.47	0.15	1.02	2.03	1.43	0.58	-1.09	0.44	0.53	1.42	0.17	0.05
**STAT1**	7.91	2.32	6.09	1.65	-1.66	0.37	-1.05	0.33	4.3	1.11	-1.35	0.09
**STAT2**	3.5	0.45	4.9	1.8	1.14	0.07	1.25	0.29	1.74	0.34	1.195	1.74
**MDA5**	58.97	14.54	50.21	18.8	-1	0.09	1.2	0.21	6.43	0.133	-1.35	0.33
**IL12A**	3.2	0.58	3.11	0.7	-1.2	0.152	1.31	0.49	1.53	0.32	1.995	0.03
**IL12B**	3.59	1.85	2.77	3.6	5.13	0.99	-2.14	1.26	-0.95	1.99	0.36	1.97
**GBP1**	17.13	1.87	32	3.33	-1.86	0.65	2.46	2.1	2.14	0.21	-1.43	0.28
**TLR8**	0.22	1.48	-1.11	0.38	-1.2	0.095	-1.08	0.5	-0.5	1.46	1.25	0.08
**TLR7**	-0.28	2.72	-1.11	1.54	1.42	0.59	2.36	0.99	1.23	0.375	1.22	1.85
**IRF7**	50.17	17.1	80.1	21.03	2.24	1.78	-1.01	0.24	6.72	0.71	0.615	0.19
**IFNβ**	**15511**	**10160**	**10594.5**	**2207.8**	8.3	1.29	3.01	0.38	35.4	9.84	5.65	3.15
**NFκB1**	3.85	0.74	-1.23	0.28	2.66	1.08	1.41	0.38	1.35	0.34	2.035	0.268
**TLR9**	1.78	0.468	-1.38	0.31	-1.22	0.11	-1.14	0.75	-0.43	1.72	-1.18	0.05
**TNF[illegible]**	**315.16**	**208.4**	**2.4**	**0.09**	3.37	2.01	2.8	1.1	20.52	23.66	3.085	0.127
**IFI6**	84.58	33.08	43.26	20.95	4.7	0.45	1.07	0.22	15.99	9.58	2.885	1.56
**IFI27**	790.16	88.9	2.9	0.7	3.53	0.98	-1.07	0.36	34.93	5.91	1.23	1.44
**IRF1**	6	0.06	2.99	0.71	1.65	0.74	1.21	0.69	1.71	0.39	3.18	0.9

**ISG20**	310.35	30.33	0.255	2.04	-1.08	0.036	-1.04	0.55	4.38	0.82	-1.12	0.4
**OAS1**	2949	2062.14	229.93	187.4	9.46	1.15	1.98	1.05	81.41	7.31	1.77	0.32
**RSAD2**	3637.4	3121.22	49.65	35.92	3.64	0.72	1.26	1.28	66.37	7.59	1.65	0.09
**RIGI**	86.34	17.71	5.92	0.1	2.51	1.55	1.51	0.41	7.36	2	-1.1	0.4
**IL18**	1.29	0.1	-1.9	0.7	-3.1	1.6	1.18	0.76	-0.45	1.28	-1.41	0.09
**IL8**	**565**	**100.26**	**-1.49**	**0.36**	2.89	1.11	1.84	0.19	27.25	8.07	1.39	0.36
**IL1β**	**112.3**	**14.31**	**3.16**	**2.95**	2.31	0.95	16.29	3.6	8.42	3.8	0.08	0.06
**MX1**	632.33	200.61	44.32	4.24	0.38	2.55	1.79	0.44	37.45	6.53	-1.65	7
**TLR3**	15.26	6.8	4.37	0.74	0.57	1.53	1.84	1.1	2.72	0.27	-1.065	0.04

Supplemental Table S3. c-Rel transcripts were detected in several big brown bat tissues by PCR.

**Big brown bat organ**	**c-Rel transcript**
Spleen	+
Gut	+
Ileum	+
Kidney	+
Lung	+
Liver	+

Efk3 cell line	+

Supplemental Table S4. Accession numbers, primer sequences and annealing temperatures for human and big brown bat genes and primers.

**Gene**	**NCBI Accession - Human**	**NCBI accession *E. fuscus***	**Primers - human (forward and reverse)**	**Annealing temperature**	**Primers– *E. fuscus* (forward and reverse)**	**Annealing temperature**
**IFNβ**	NM_002176.3	XM_008145044.1	GCTTGGATTCCTACAAAGAAGCA ; ATAGATGGTCAATGCGGCGTC	54	GCTCCGATTCCGACAGAGAAGCA ; ATGCATGACCACCATGGCTTC	56
**NFκB - 1**	NM_003998.3	XM_008156644.1	GAAGCACGAATGACAGAGGC ; GCTTGGCGGATTAGCTCTTTT	54	GAAGCACGGATGACAGATGC ; GCCTGGCGGATGATCTCCTTT	56
**IL12A**	NM_000882.3	XM_008142364.1	CCTTGCACTTCTGAAGAGATTGA ; ACAGGGCCATCATAAAAGAGGT	53	TCCTGCACTTCTGAAGAGATTGA ; ACAGGGTCGTCATAAAAGAGGC	52
**IL12B**	NM_002187.2	XM_008147661.1	ACCCTGACCATCCAAGTCAAA ; TTGGCCTCGCATCTTAGAAAG	55	ACCTTGACCATCCAAGTCAAA ; TTTGCTTCACATTTTAGAAAG	52
**IRF3**	NM_001571.5	XM_008154348.1	AGAGGCTCGTGATGGTCAAG ; AGGTCCACAGTATTCTCCAGG	55	AGAAGCTAGTGATGGTCAAG ; AGGTCCACAGTGTTCTCCAGC	56
**IL18**	NM_001562.3	XM_008150175.1	TCTTCATTGACCAAGGAAATCGG ; TCCGGGGTGCATTATCTC	51	TCTTCGTTAACCAGGGAAGCCAA ; TCTGGGGTGCATTCTCTTC	52

			TAC		AC	
**IRF7**	NM_001572.3	XM_008159582.1	CCCACGCTATACCATCTACCT ; GATGTCGTCATAGAGGCTGTTG	57	CCCGCACTGCACCATCTACCT ; CAGGTCCTCGTACAGGCTGTTG	56
**STAT1**	NM_007315.3	XM_008146505.1	CAGCTTGACTCAAAATTCCTGGA ; TGAAGATTACGCTTGCTTTTCCT	54	CAGCTGGACGCCAAGTTCCTGGA ; TGCAGGTTGCGCTTGCTCTTCCT	56
**STAT2**	NM_005419.3	XM_008148645.1	GAGCCAGCAACATGAGATTGA ; GCCTGGATCTTATATCGGAAGCA	53	GAGCCAGCAGCATGAGATCGA ; GCCTGAGTCTTGTATCGGAAGAG	56
**TLR3**	NM_003265.2	XM_008151907.1	TCAACTCAGAAGATTACCAGCCG ; AGTTCAGTCAAATTCGTGCAGAA	53	TCAGCTCAGAAAATTACCGCCTG ; AGTTCAGTCAAATTCCGGCAGAA	52
**TLR7**	NM_016562.3	XM_008156577.1	TCGTGGACTGCACAGACAAG ; GGTATGTGGTTAATGGTGAGGGT	51	TTGTGGACTGCACGGACAAG ; GGTATGTGGTTGATGGTGAGGGT	52
**TLR8**	NM_138636.5	XM_008156576.1	GACTACAGGAAGTTCCCCAAAC ; ATACCGGGATTTCCGTTCTGG	51	GGCTGCAAGAAGTCCCCCAAAC ; TTGCAATAATTCTCACAGTAG	52
**TLR9**	NM_017442.3	XM_008155066.1	CTGCCACATGACCATCGAG ; GGACAGGGATATGAGGGATTTGG	55	CTGCCACATGACCATCGAG ; GGCCAGGGTCCGGAGGGCGGGGG	56
**TNF[illegible]**	NM_000594.3	XM_008157360.1	CAGCCTCTTCTCCTTCCTGA ; AGATGATCTGACTGCCTGGG	57	GCCCATGTTGTAGCAAACC ; GCCCTTGAAGAGGACCTGGG	56
**IL8**	NM_0005	XM_0144	ACTGAGAGTGATTGAGA	51	AAACATGACTTCCAAGCT	52

	84.3	60599.1	GTGGAC ; AACCCTCTGCACCCAGTTTTC		GG ; TGTGGTCCACTCTCAATCAC	
**IL1β**	NM_000576.2	XM_008160259.1	ATGATGGCTTATTACAGTGGCAA ; GTCGGAGATTCGTAGCTGGA	54	ATGATGGCTTACTACAGTGACAA ; GTCGGAGATTTTCAGCTGGA	52
**GBP1**	NM_002053.2	XM_008157793.1	AGGAGTTCCTTCAAAGATGTGGA ; GCAACTGGACCCTGTCGTT	54	TTCAGCTGACTTTGTGAGCT ; ACTGCTGATGGCATTAACAT	52
**IFI6**	NM_002038.3	XM_008148008.1	GGTCTGCGATCCTGAATGGG ; TCACTATCGAGATACTTGTGGGT	57	GGTCGGCCATAGCGAACGGG ; TTGTTGTCTATCTGCCTGTGGAC	56
**IFI27**	NM_206949.2	XM_008159947.1	TGCTCTCACCTCATCAGCAGT ; CACAACTCCTCCAATCACAACT	56	GCCAAGATGATGTCATCAGC ; CACATCCAGGCCCCAATAG	56
**IRF1**	NM_002198.2	XM_008142488.1	ATGCCCATCACTCGGATGC ; CCCTGCTTTGTATCGGCCTG	56	ATGCCCATCACTCGGATGC ; CCCGACTTTGTACCGGCCTG	56
**ISG20**	NM_002201.5	XM_008155878.1	TCTACGACACGTCCACTGACA ; CTGTTCTGGATGCTCTTGTGC	56	CCCGAGACACCTCCCATATTC ; CCAGCCTGGATGTCCCGGTTC	56
**MX1**	NM_001144925.2	XM_008145691.1	AGGACTACGAGATTGAGAT ; TTATGCCAGGAAGGTCTA	51	GGACATGGAGATCAATCTT ; TGATGCCAGGAAGGTCTA	52
**OAS1**	NM_016816.3	XM_008142919.1	AGTTGACTGGCGGCTATAAAC ; GTGCTTGACTAGGCGGA	55	AGGTGACGGACGACTACAGAC ; GTGCTTGACCAGGCGGAT	56

			TGAG		GAG	
**RSAD2**	NM_080657.4	XM_008162430.1	TGGGTGCTTACACCTGCTG ; GAAGTGATAGTTGACGCTGGTT	53	CGAGTACCTGGGCCGCCTG ; GAAGCGGTTGATGACGGAGTTG	56
**RIG-I**	NM_014314.3	XM_008142005.1	CTGGACCCTACCTACATCCTG ; GGCATCCAAAAAGCCACGG	55	CTGGACCCCACCTACGTCCTC ; AGCATCCAAAAAGCCACGA	56
**MDA5**	NM_022168.3	XM_008138631.1	GCCCGCTACATGAACCCTG ; CAGCAATCCGGTTTCTGTCTT	55	GCCCGCTACATGGACCCCG ; CAGAAACCCGACTTCTGTCTT	56
**GAPDH**	NM_002046.5	XM_008144826.1	GGAGCGAGATCCCTCCAAAAT ; GGCTGTTGTCATACTTCTCATGG	57	GGAGCGAGATCCCGCCAACAT ; GGGAGTTGTCATACTTGTCATGG	56
**β-actin**	NM_001017992.3	XM_008156007.1	CTCGACACCAGGGCGTTATG ; CCACTCCATGCTCGATAGGAT	57	CCCGGCACCAGGGCGTGATG ; CGATGCCGTGCTCGATGGGGT	56
**TLR2**	NM_003264.4	XM_008144148.1	TTATCCAGCACACGAATACACAG ; AGGCATCTGGTAGAGTCATCAA	52	TTATCCAATACAAGAATACAGAG ; AGGCATCTGGTAGAGTCTTCAA	51
**c-Rel**	-	XM_008162099.1	-		GACGACTACTCTACCTCCTG ; ATCTTCACCGTCACTGGCTC	56
**β2-microglobulin**	NM_004048.2	-	GAGGCTATCCAGCGTACTCCA ; CGGCAGGCATACTCATCTTTT	55	-	

Supplemental Table S5. siRNA sequences designed against *E. fuscus* (big brown bat) TLR3, RIGI and MDA5. r = ribose sugar; BBB = big brown bat.

**siRNA sequence**	**Duplex name**	**siRNA duplex sequences**	**Binding position on transcript**
BBB TLR3 siRNA sequence	CD.Ri.26282.13.2	5' rCrUrArUrCrUrCrArArCrUrUrUrCrUrGrArCrArArArArCCT 3'	BBB TLR3 - position 337-362
BBB TLR3 siRNA sequence2		5' rArGrGrUrUrUrUrGrUrCrArGrArArArGrUrUrGrArGrArUrArGrCrU 3'	
BBB TLR3 siRNA sequence	CD.Ri.26282.13.1	5' rCrArArCrArUrGrArGrArUrArCrCrUrGrArArUrUrUrArAGA 3'	BBB TLR3 - position 972-997
BBB TLR3 siRNA sequence2		5' rUrCrUrUrArArArUrUrCrArGrGrUrArUrCrUrCrArUrGrUrUrGrArA 3'	
BBB RIGI siRNA sequence	CD.Ri.26280.13.2	5' rArGrCrUrUrUrCrUrArArArCrCrUrGrArGrArUrArUrUGA 3'	BBB RIGI - position 1972-1997
BBB RIGI siRNA sequence2		5' rUrCrArArUrArUrCrUrCrArGrGrUrUrUrArGrArArArGrCrUrGrA 3'	
BBB RIGI siRNA sequence	CD.Ri.26280.13.10	5' rGrUrGrGrCrArArCrArGrUrCrArArArGrArCrArArCrUrUGG 3'	BBB RIGI - position 1312-1337
BBB RIGI siRNA sequence2		5' rCrCrArArGrUrUrGrUrCrUrUrUrGrArCrUrGrUrUrGrCrCrArCrCrA 3'	
BBB MDA5 siRNA sequence	CD.Ri.26279.13.2	5' rArCrUrUrGrGrArCrArArGrArArArArArArGrCrArUrCrUGA 3'	BBB MDA5 - position 1256-1281
BBB MDA5 siRNA sequence2		5' rUrCrArGrArUrGrCrUrUrUrUrUrUrCrUrUrGrUrCrCrArArGrUrGrA 3'	
BBB MDA5 siRNA sequence	CD.Ri.26279.13.1	5' rGrArArGrArUrUrCrUrUrUrUrArArArArCrArUrUrGrUrCAA 3'	BBB MDA5 - position 3301-3326
BBB MDA5 siRNA		5'	

sequence2		rUrUrGrArCrArArUrGrUrUrUrArArArArGrArArUrCrUrUrCrArA 3'	

Supplementary Methods

c-Rel Western Blots: HEK293T cells were seeded at a concentration of 3x 10^5 cells/well in six well plates and simultaneously transfected with 100nM of 1:1 cocktail of two different siRNA specific to c-Rel (table 1), NC siRNA or no siRNA and plasmids expressing HA-tagged c-Rel. Cells were harvested in sample buffer for western blots 24 hrs post transfections. Western blots were carried out as previously mentioned [2]. Primary antibodies used were: 1: 1,000 mouse anti-HA (Clone HA7; Sigma) and 1: 5,000 rabbit anti-GAPDH (Cedarlane). Secondary antibodies used were: 1:10,000 goat anti-mouse Alexa 488 (Molecular Probes) and 1: 10,000 goat anti-rabbit Cy5 (GE Healthcare).

TLR3, RIGI and MDA5 knockdown: Dicer-ready siRNA (DsiRNA) specific to big brown bat TLR3, RIGI and MDA5 were designed and obtained through Integrated DNA Technologies (IDT). A 100nM final concentration of a 1:1 mixture of two DsiRNAs (see Supplementary Table S5) targeting separate regions on the respective transcripts was transfected into Efk3 cells using Lipofectamine 2000. Scrambled non-specific DsiRNA (NC DsiRNA; IDT) was used as a negative control. Transfections were carried out as previously mentioned for siRNA [3]. In brief, cells were transfected with the siRNA cocktail for 24 hours and then transfected with 750ng/ml poly(I:C) using Lipofectamine 2000. RNA was extracted 16h after poly(I:C) stimulation for cDNA preparation and qRT-PCR.

References:

1 Zhang, G. *et al.* Comparative analysis of bat genomes provides insight into the evolution of flight and immunity. *Science* **339**, 456-460, doi:10.1126/science.1230835 (2013).

2 Bergeron, T. *et al.* The effect of Zhangfei on the unfolded protein response and growth of cells derived from canine and human osteosarcomas. *Vet Comp Oncol* **11**, 140-150, doi:10.1111/j.1476-5829.2011.00310.x (2013).

3 Nasirudeen, A. M. *et al.* RIG-I, MDA5 and TLR3 synergistically play an important role in restriction of dengue virus infection. *PLoS Negl Trop Dis* **5**, e926, doi:10.1371/journal.pntd.0000926 (2011).
